# An Analysis of the Severity of Food Safety Hazards in EU Food Fraud Cases

**DOI:** 10.3390/foods14244328

**Published:** 2025-12-16

**Authors:** Martin Alewijn, Pauline Goemans, Karen E. Gussow, Kate J. Turner, Annemieke M. Pustjens

**Affiliations:** 1 Wageningen Food Safety Research (WFSR), Wageningen University and Research, P.O. Box 230, 6700 AE Wageningen, The Netherlandsannemieke.pustjens@wur.nl (A.M.P.); 2 Netherlands Food and Consumer Product Safety Authority-Intelligence and Investigation Service (NVWA-IOD), P.O. Box 43006, 3540 AA Utrecht, The Netherlands; p.goemans@nvwa.nl (P.G.); k.e.gussow@nvwa.nl (K.E.G.)

**Keywords:** food fraud, food safety, media reports, criminal investigations, Europe, classification, food laundering

## Abstract

We aim to evaluate the extent to which food fraud incidents cause food safety concerns, using three complementing sources: (1) the publicly available fraud issues as collected by the Joint Research Centre (JRC) of the European Union in monthly food fraud reports; (2) academic literature that documents food fraud incidents; and (3) reports of criminal investigations of food fraud in The Netherlands. We studied the nature of these concerns in terms of their severity, types of hazard and investigate this in relation to the type of food fraud as well as the types of product. The findings reveal that approximately one out of three cases of food fraud carries a considerable food safety risk. Within these cases, food laundering—(re)introducing already illegal food into the food chain—was the main type of fraud most predominant and carried most food safety risk. This study demonstrates how food frauds have further-reaching food safety consequences than meets the eye, impacting on the food safety system. Perpetrators, amongst other flaws, seem to consciously ignore food safety risks and regulations. The analytical research community could consider not focusing exclusively on fraudulent food enhancement, but also consider how to extend their contribution to the detection of food laundering.

## 1. Introduction

The impact of food fraud on food safety has been gaining recognition in recent decades, informed by a number of notable incidents. Of particular importance for the recent increased attention levels was the incident in China in 2008 when plasticizer melamine was used to deceive protein tests on dairy, which led to the death of six babies and the illness of hundreds of thousands of infants in China [1]. Consequently, the EU banned imports of baby food containing Chinese milk, and called for more checks on other Chinese food imports [2]. The incident led to consumers distrust the (Chinese) dairy industry and caused a major shift in demand from more trusted countries. In addition to the impact on food safety, the fraud was also devastating for consumers’ trust in the food system and had far-reaching economic consequences. Though traditionally food safety had been regarded as an unintentional event, this fraud with global consequences drew the attention of food regulators to the phenomenon of food fraud. Notably, the General Food Law was established the European Union in 2002 [3]. This law is the foundation of food and feed law and lays down general principles, requirements and procedures with respect to food and feed safety, covering all stages of food and feed production and distribution. Many other regulations, directives and decisions are in place for various aspects of food safety and food fraud. Although food fraud is not explicitly defined in EU legislation, instances of fraudulent practices are addressed indirectly through general consumer-protection and food-safety laws. For example, the Food Information to Consumers Regulation [4], and the Official Controls Regulation [5], aim to prevent misleading practices and protect consumers—a focus that has also been underlined by Ulberth [6].

**Figure 1:**
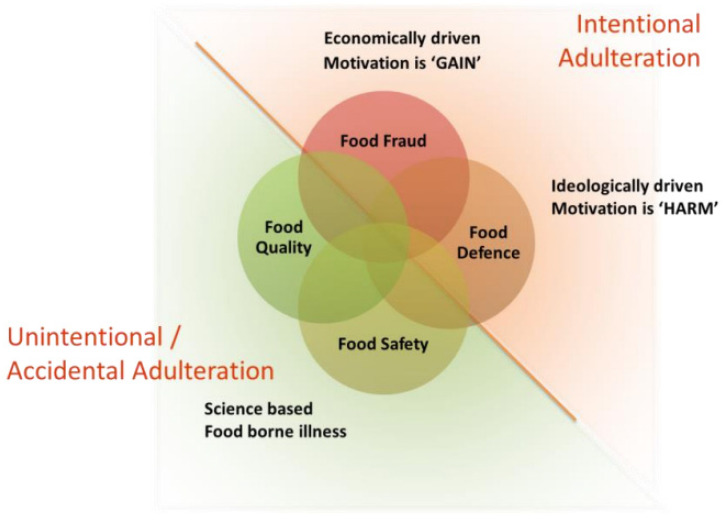
Schematic relations between food fraud, food defence, food safety, and food quality. Taken from GFSI [8].

An influential study by Spink & Moyer [7] pointed out the potential effects on food safety that food fraud could cause, even if the intent of food fraud is usually economic or financial. This difference is visualized in Figure 1. This illustrates that incidents of food fraud, food safety, food quality, and food defence can be distinguished based on their intentionality and motivational background. Food fraud is economically driven and oriented towards obtaining gain; food terrorism is ideologically driven and aimed at causing harm. Though this figure depicts a food safety incident as largely unintentional, it also clearly shows an overlap between food fraud and food safety. The extent and nature to which food fraud and food safety overlap have however not yet been explicitly studied.

In addition, the Spink & Moyer study [7] formulated a definition of food fraud that was adopted by the Global Food Safety Initiative (GFSI) and is still widely used. It defines food fraud as follows “A collective term encompassing the deliberate and intentional substitution, addition, tampering or misrepresentation of food, food ingredients or food packaging, labelling, product information or false or misleading statements made about a product for economic gain that could impact consumer health” [7]. Subsequent research has broadened this perspective. Everstine et al. showed that economically motivated adulteration often exploits gaps in traditional food-safety controls and can pose significant health risks [9]. Lord et al. emphasized that food fraud arises from systemic and situational factors within supply chains, not just from individual offenders [10]. Manning & Soon highlighted persistent inconsistencies in terminology and argued that intentional, economically driven adulteration requires distinct risk-assessment and prevention approaches [11]. Together, these studies build on and refine the Spink & Moyer definition by examining the wider contextual, regulatory, and risk-based dimensions of food fraud.

Gussow & Mariet [12] discussed a number of limitations of this leading definition as well as of other approaches to capturing the concept of food fraud and claim these are unsatisfactory to comprehensively analyse the phenomenon. In particular, the authors emphasize the occurrence of food laundering. They argue, based on over 50 cases of food fraud that have been investigated by the Dutch authorities between 2010 and 2018, that an equally large part of the deceitful behaviour with regard to food is not to do with fraudulently enhancing the value of food by substitution, tampering, misrepresentation, or false statements about a food product. Rather, this is aimed at bringing, keeping, or reintroducing illegal food into the food supply chain, such as depreciated foodstuffs with chemicals exceeding acceptable limits, animal byproducts not fit for human consumption, or illegally produced food [12]. This intended outcome is not expressed in the food fraud definitions to date. They have suggested the definition of food fraud as follows: “Food fraud is committed by any actor who is intentionally involved in illegal acts for economic advantage, thus causing or facilitating illegal food to be laundered into the supply chain or for food to be fraudulently value-enhanced”. However, further research is needed to test this definition on a broader set of food fraud incidents and outside of the Dutch context.

In addition, the focus of the academic studies on defining food fraud has largely drifted away from the central message of the influential study of Spink & Moyer [7]; that is, to research and determine the food safety threat of food fraud. Most studies acknowledge the unexpected food safety risks caused by fraudulent and deceitful behaviour, which is typically designed to avoid detection and circumvent regulatory food safety systems (e.g., refs. [9,13,14]), yet go on to interpret the patterns and types of food fraud rather than researching the actual associated food safety threats. Similarly, academic literature concerning analytical method development often uses examples of incidents to justify the importance of authenticity, but there are no studies to date, to our knowledge, that focus on the nature and scale of the food safety threats. This would, however, be very useful to prioritize and focus the research on analytical method development and where to apply controls, with the potential to prioritise the detection of food fraud with associated health concerns. Finally, examples of food safety threats caused by food fraud often draw from examples worldwide, such as the melamine incident in China. Although the food supply chain is a global matter, it would be insightful to understand the food safety threat in Europe more specifically. This could also assist the work of the European Food Fraud Network (The EU Agri-Food Fraud Network—European Commission) and guide future research on analytical methods.

The aim of this article is therefore twofold. First and foremost, it seeks to evaluate the extent to which food fraud incidents cause food safety concerns. We study the nature of these concerns in terms of their severity and types of hazard and investigate this in relation to the type of food fraud as well as the types of product. The goal is to arrive at meaningful results about the nature of food fraud in relation to food safety threats, which could guide future research to develop methods to detect these issues. A secondary aim is to test the definition of food fraud as suggested by Gussow & Mariet [12] outside of the Dutch context, also connecting the safety concerns to these novel definitions. We do this by using a broader dataset of food fraud incidents that occurred in Europe using three different sources: (1) the publicly available fraud issues in Europe as collected by the Joint Research Centre (JRC) of the European Union from September 2016 to March 2023; (2) academic literature that documents European food fraud incidents; and (3) reports of criminal investigations of food fraud in The Netherlands carried out in the period 2016–2023 for additional qualitative and in-depth observations. In addition, we elaborate on the novel conceptualization of food fraud, drawing from categories developed by ACN (Alert and Cooperation Network) and CEN (European Committee for Standardization), to devise a more robust classification that we expect to yield meaningful insights with regard to food safety. Lastly, as food fraud prevention measures are diverse in different parts of the world, we aim to focus on the EU, which is seen as an example of a region with relatively high levels of regulation and control, warranted by the EU policy and activities to combat food fraud.

## 2. Approach

Three main data sources of incidences of food fraud issues were used: (1) the Monthly Food Fraud Summary Reports of articles (grey literature) published by the Joint Research Centre (JRC) food fraud cases in the European Union (EU); (2) academic publications which are written within or concern the EU; and (3) reports of criminal investigations of food fraud in The Netherlands. Details are specified in Section 2.1, Section 2.2 and Section 2.3. We excluded a number of other potentially interesting food fraud databases, such as HorizonScan, FoodChainID Food Fraud Database (formerly known as Decernis), RASFF, and MedISys for Food Fraud. These databases were not included in the current study because they are not all publicly accessible, or they focus on the United States of America and not on Europe. Also, some databases report issues where fraud is not confirmed, whereas our focus is on gathering insights into actual occurrences of food safety risks in relation to food fraud.

### 2.1. JRC Monthly Food Fraud Summary Reports

JRC is the Joint Research Centre of the European Commission. They publish Monthly Food Fraud Summary Reports, which are publicly available online [15]. The food fraud cases in these summaries are sourced from media reports selected from relevant sources, such as the Medical Information System (MedISys [16]) and RASFF [17]. The JRC includes cases of food fraud that meet the four criteria that the European Commission has formulated for food fraud: (1) Violation of EU food law; (2) Intentional fraud; (3) Economic motivation; (4) Customer deception. The fraud must have a significant impact, and priority is given to fraud that causes a public health risk; however, safety concern is not a criterion for reporting [18]. All available JRC Monthly Food Fraud Summary Reports at the time of sourcing the literature were identified and analysed for the review, which included food fraud cases from September 2016 to March 2023. Food fraud cases in the JRC Monthly Food Fraud Summary Reports were filtered on whether they concerned EU countries. Only EU-related food fraud cases were considered relevant.

### 2.2. Academic Literature

A review was performed based on ‘Good review practice: A researcher guide to systematic review methodology in the sciences of food and health’ [19]. To investigate the relationship between food fraud and food safety mentioned in the literature, the key elements of the following research question were determined: What does the academic literature in the EU indicate about the extent of food safety risks related to food fraud? The covered elements included food, food fraud, food safety risk, health risk, and the EU. The titles and abstracts of relevant academic publications were mined to build the search strategy. Four academic databases, namely, Food Science and Technology Abstracts (FSTA), PubMed, Web of Science Core Collection, and Scopus, were searched for relevant academic publications on food fraud safety risks. For each database, an individual search string was created as shown in Supplementary Table S1. To prevent the exclusion of historical food fraud and safety cases, searches were conducted with no date limitation. As presented in Supplementary Table S2, strict eligibility criteria were used during the search process so that the number of relevant academic publications was feasible to review within the available timeframe. Due to the difficulty in dividing EU studies pre- and post-Brexit, the screening process excluded publications concerning the United Kingdom, although this country was initially included in the search string for FSTA. Cases involving either the EU explicitly or where the region was not specified were included. Food fraud examples mentioned in the academic publications that did not meet these criteria were not seen as relevant to this review.

### 2.3. Criminal Investigation Reports of Food Fraud in The Netherlands

The enforcement authority for food safety in the Netherlands is the Netherlands Food and Consumer Product Safety Authority (NVWA). The Intelligence and Investigation Service of the Netherlands Food and Consumer Product Safety Authority (NVWA-IOD) is tasked with performing criminal investigations with regard to food fraud. The unit has police investigative capabilities, which it uses under the authority of the Dutch Public Prosecution Service. The Netherlands Food and Consumer Product Safety Authority works nationally and covers the full food supply chain, supervising regulatory areas from farm to fork. This includes the primary production of animals and crops, feed producers, imports and exports, slaughterhouses and cutting plants, food producers, retail, and catering businesses. Therefore, the criminal investigations of the Intelligence and Investigation Service cover food fraud incidents throughout various supply chains and have a national reach. All food fraud criminal investigations carried out in the period 2016–2023 by this unit, and for which a criminal report has been submitted to the Public Prosecution Service, have been selected for this study. The food safety effects of each of these food fraud incidents have been qualitatively analysed using data from the criminal report, internal reports of the NVWA-IOD, media coverage, and verdicts.

### 2.4. Analysis

**Figure 2:**
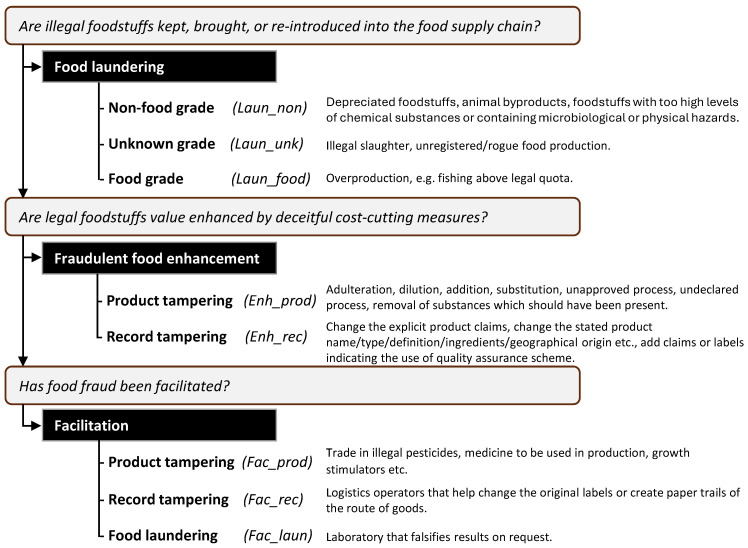
Flow chart used to categorise the main and subtypes of food fraud in this study, including some descriptive examples for each subtype.

The food fraud incidents that were retrieved have been categorized into four levels:

1. Risk category. The magnitude of the hazard is indicated by a four-point scale representing the reported (or deduced) food safety issue resulting from a food fraud issue:
  - Category A. Cases where an actual hazard is present in the food AND the resulting injuries or harm to consumers have been reported;
  - Category B. Cases where an actual hazard is present in the food and reported in levels where food safety risks are likely, BUT no injuries have been reported;
  - Category C. Cases where it is likely that fraudulent actions could result in an elevated risk of food hazards being present in the food, BUT the actual presence of hazards has not been demonstrated or reported;
  - Category D. Cases where the resulting product is considered safe to eat, although unfair practices have been observed and product traceability is problematic.
2. Hazard type. In case a food hazard was reported, the food hazard was classified as either allergenic, biological, chemical, or physical:
  Al.—Allergenic: The unintended or unlabelled presence of one or more allergens in a food product. Symptoms of allergic reactions include itchiness, swelling of the throat and mouth, difficulty breathing, and potentially death. In the EU, fourteen food allergens are recognized: molluscs, eggs, fish, peanuts, soybeans, milk, gluten, crustaceans, mustard, nuts, lupin, celery, sulphites, and sesame.
  Bi.—Biological: Contaminants that stem from microorganisms that can cause a food safety risk. Examples are viruses, bacteria, moulds, and yeasts.
  Ch.—Chemical: Chemical substances that occur naturally in foods (e.g., tetrodotoxin in pufferfish and saponins from legumes) or are added to foods (e.g., unauthorized food additives, preservatives, food contact materials, cleaning agents, and agricultural chemical residues) that can result in illness or injury.
  Ph.—Physical: Foreign objects or materials from both natural (e.g., fruit stems, small stones) and unnatural (e.g., plastic or metal fragments, screws, glass shards) sources that can either cause injury or create conditions favourable for the growth of pathogens.
3. Food fraud category. Food fraud cases were classified according to the decision model in Figure 2, which was developed for this study. Note that it will yield a primary food fraud category, which has been used in this paper; however, by answering all questions, one or more additional categories may be applicable to real-life cases. The categorisation model uses the main categories from Gussow and Mariët [12] and is further subcategorized from ACN notifications from the European Commission [20], with the distinction between physical and administrative actions coming from the definitions of food fraud in CEN 17972:2024 [21]. The model was developed after the dataset was initially classified based on the food fraud types used by Winkler et al. [22], which included document forgery, adulteration, grey market activities, counterfeit, and misdescription/mislabelling/misbranding. However, the preliminary results demonstrated that these types were not distinctive enough. For example, we could not properly distinguish between misbranding and counterfeit or to what extent products are tampered with in counterfeit or grey market activities. This resonated with the arguments made in Gussow & Mariet [12]. Furthermore, we considered it important to distinguish between physical tampering with products versus record tampering when investigating the associated risks and hazards. Therefore, we added this subclassification to fraudulent food enhancement. The concept of food laundering refers to materials that are illegal to sell as food. ‘Illegal’, however, does not necessarily imply unsafe or unfit food. For example, an endangered fish species may be illegal to catch but safe to consume, whereas nuts with aflatoxins are both illegal to sell and unsafe to consume. Therefore, it is specified whether these illegal materials were food grade, of an unknown food grade, or of non-food grade.
4. Product category. Product categories as used by RASFF [17] were used, with three modifications: (1) ‘wine’ and ‘alcoholic beverages’ were merged to ‘alcoholic beverages and wine’; (2) ‘bivalve molluscs and products thereof’, ‘cephalopods and products thereof’, ‘crustaceans and products thereof,’ and ‘fish and fish products’, were merged to ‘aquatic foods and products thereof’; (3) ‘meat and meat products (other than poultry)’ and ‘poultry meat and poultry meat products’ were merged to ‘meat and meat products’. Many news items mentioned in the JRC Monthly Summary Reports report cases involving multiple food products. For these items, the individual food products were taken into account as individual food fraud records. In some cases, the individual products were not specified; those are reported in the category ‘other food product/mixed’.

Cases were counted and visualised as clusters of stacked bar plots, using a custom script in R (R 4.4.1, R Foundation for Statistical Computing, Vienna, Austria).

### 2.5. Data Source Representativeness

All three data sources have their strengths and also some salient weaknesses. They are, however, similar in the fact that each source is limited to detected food fraud cases. By definition, perpetrators of food fraud intend to hide their actions, and in all likelihood, only a fraction of cases are actually reported (as previously suggested by others [1,6,14,23]). Similarly, in published cases some (additional) hazards may have been unnoticed and consequently unreported. In addition, the sources consulted all have their own specific biases.

The JRC Monthly Food Fraud Summary Reports provide a good volume of cases, which is good for external validity. However, it is limited to detected cases that have been mentioned in a media or RASSF report and could be skewed towards countries with more active enforcement. For example, Portugal (Economic and Food Safety Authority (ASAE)), Italy (Anti-adulteration and healthcare unit (NAS) of the Carabinieri), and Spain (Guardia Civil) have active food fraud units. Some over-representation within the JRC of the more active countries, especially if they also have a more active press strategy, might be possible. Also, risk analysis of specific food categories may be biased towards region-specific fraud types, lacking EU-wide universality.

Academic papers include studies of enforcement data that complement the media focus, improving the internal validity. Although academic papers commonly aim to present scientific novelties, non-review papers in particular tend to focus on the more impactful cases rather than providing comprehensive coverage of known cases of food fraud or on results of routine analyses. Therefore, we anticipate some bias in the academic literature towards more severe cases of food fraud. Most researchers in the academic literature have focused on various analytical techniques used to identify or quantify adulterants and contaminants in food, such as the identification of species substitution and quantification of unapproved chemicals [24,25,26,27]. It is worth noting that there is some overlap, as some papers refer to the same incidents.

The data from the NVWA-IOD concern cases for which a criminal report has been submitted to the Dutch Public Prosecution Service. The data is limited in terms of volume and concerns merely one European country. The unit prioritizes food fraud cases with (potential) negative effects on food safety. The prosecution of these investigations is not yet finalized in all instances, and some investigations may have been dismissed for technical or policy reasons. Some of the food fraud incidents are thus technically alleged food frauds. Still, the criminal report with collated evidence about the alleged fraud is sufficient for discussing and analysing the relation between the alleged fraud and food safety. This data source provides the most empirically in-depth material on food fraud. A cross-check with the JRC cases underlines the complementary nature, as only two cases appeared in the JRC dataset. This source, consisting of reports from criminal investigations, also enabled some observations on the behaviour of food fraudsters, particularly where such behaviour impacts food safety.

Overall, the use of three sources that complement each other ensures a level of triangulation that is positive for both internal and external validity. It is also valid to assume that the safety concerns observed in the collective cases are real and offer an insight into the occurrence of food fraud, its safety concerns, and some consequences and drivers from three different perspectives.

## 3. Results

### 3.1. General Observations

The query of the JRC Monthly Food Fraud Summary Reports between 2016 and 2023 yielded 659 cases of EU-associated food fraud (referred to as ‘JRC cases’ in this paper; Supplementary Figure S1). About one third, 216 cases, fall into risk categories A–C and are considered to be a safety risk, and about 11% (73 cases) have a confirmed hazardous issue (categories A–B).

The academic literature yielded 105 cases up to March 2023 (Supplementary Figure S2), all of which were classified into risk categories A–C, and almost all (96 cases) into the confirmed hazardous categories (A–B). Of the 31 criminal investigation reports from the NVWA-IOD on food fraud that have been included in this study, about 75% (24 cases) have been classified as risk categories A–C and have a certain safety risk. About 50% (16 cases) have a confirmed hazardous issue (categories A–B). The NVWA-IOD investigated five food fraud cases associated with health issues (category A), in which reports were submitted to the food safety authority regarding consumers falling ill or sustaining injuries from food products. The cases from the NVWA-IOD are proportionally more severe than the JRC cases, with academic literature showing an even stronger focus on high-severity incidents. All individual cases are described and classified in Supplementary Table S3 (JRC data), Supplementary Table S4 (Academic literature), and Supplementary Table S5 (Criminal investigation reports).

**Figure 3:**
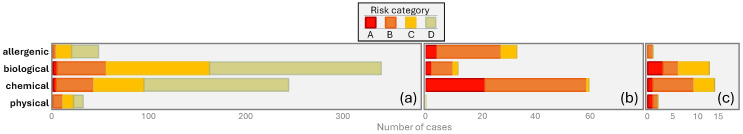
Number of reports per risk category and per hazard type, found in the (**a**) JRC reports, (**b**) academic literature, (**c**) NVWA-IOD cases.

**Figure 4:**
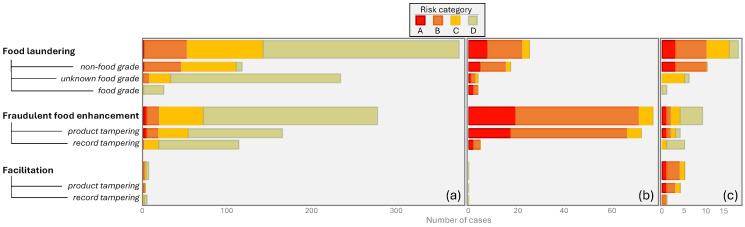
Number of reports per risk category and per fraud type category, found in the (**a**) JRC reports, (**b**) academic literature, (**c**) NVWA-IOD cases.

**Figure 5:**
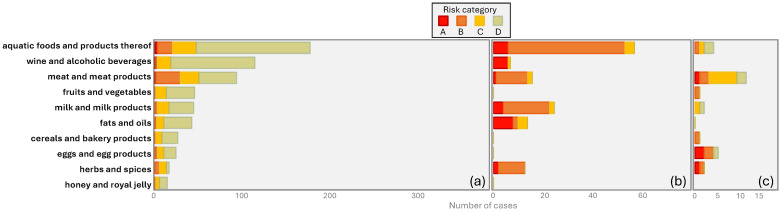
Number of reports per risk category and per food category, found in the (**a**) JRC reports, (**b**) academic literature, (**c**) NVWA-IOD cases. The top 10 food categories in the JRC data are displayed in descending order.

The safety aspects are further broken down by type of hazard, type of fraud, and food type, in Figure 3, Figure 4 and Figure 5.

### 3.2. Hazard Type

As a result of the fraudulent activity and possibly the food type at hand, food products containing one or more hazard types might be present in the food chain. Figure 3 shows the number of reports per risk category (A–D) and hazard type (allergenic, biological, chemical and physical). Some cases have no hazard specified; for example, when they deal with smuggling, relabelling, contraband products, or illegal fishing. In the JRC cases, the vast majority of hazards were of biological or chemical nature, whereas (potentially) allergenic and physical hazards were encountered far less frequently. In the academic literature, chemical hazards are dominant and, interestingly, allergens are also reported frequently. The quantitative distribution of hazard types for the NVWA-IOD cases is quite similar to the JRC’s.

### 3.3. Food Fraud Type

Figure 3 shows the number of reports per risk category (A–D) and fraud type category (as defined in the flowchart in Figure 2). In terms of fraud types, food laundering is the most abundant type encountered in the JRC reports (375 cases, 57%), followed by fraudulent food enhancement (278 cases, 42%). Facilitation cases are very few (6 cases), and although displayed in the figure, these numbers are too low to draw any other firm conclusions. In terms of risk categories, food laundering is not only the more abundant category, but it also has a higher proportion of risk B (14 versus 7%) and C (24 versus 18%) cases than food enhancement. Food enhancement has a few more cases in the most serious risk A category (4% versus 1%), but given the small numbers, it is difficult to determine if this difference is significant.

Within the laundering fraud type, the unknown food grade is the largest subcategory, non-food being second in absolute numbers. Laundering with unknown food-grade material mainly originates from a lack of traceability information. In this case, it is difficult to judge the severity level. However, it always holds a risk in case a product needs to be recalled. Unsurprisingly, risks associated with non-food-grade food laundering are very high, with almost 40% and 55% of cases in severity categories B and C, respectively. The risk for food-grade food laundering is inherently low. Noteworthy is the low absolute number of cases (24) in the latter subcategory. This makes sense, as food laundering involves illegal food, which is often illegal because it is unfit for human consumption and therefore non-food grade or of an unknown food grade—for example, when its origin is unclear. The cases where the illegal food was considered food grade were predominantly related to overfishing above the allowed quota or illegal fishing of endangered species.

Within fraudulent food enhancement, the subcategory product tampering has a higher incidence rate, and the higher risk profiles A and B happen proportionally more often than for record tampering. This is explained by the nature of record tampering, where the product itself is not altered, but existing claims are falsified.

The cases described in the academic literature (Figure 4b) are fewer (105 cases), and on average, much more serious, with almost all risk scores belonging to the A and B categories (28 and 68 cases). This substantiates the assumption that authors tend to focus on the cases with a clear (potential) health effect, leading to some reporting bias in the academic literature. Case dates and locations are not always reported in the academic literature, so it is likely that these cases come from a wider time period and are not necessarily restricted to the EU. Another striking difference compared with the JRC cases is the types of food fraud that have been reported. Fraudulent food enhancement is quantitatively dominant (about 75% of all cases), with product tampering as the dominant subcategory, representing about 94% of these cases. This can possibly be explained by the observation that many chemical/analytical methods to detect food fraud are focused on detecting adulteration/substitution of ingredients, forms of product tampering. These methods, especially if multivariate and/or untargeted in nature, may also incidentally detect record tampering or laundering cases, but are seldom designed or tested to do so. This likely is a point of attention for the scientific community, as the laundering and record tampering cases seem, based on JRC occurrence data, to occur more frequently and are no less harmful.

The fraud type profile for the NVWA-IOD cases was quite similar to the JRC data, with food laundering being the most predominant type. Fraudulent enhancement was less frequently encountered, and within that category, record tampering was slightly more dominant than in the other two data sources. The NVWA-IOD found the facilitation type of fraud markedly more than observed in JRC and the academic literature, which will be illustrated in more detail below. In terms of food safety risks, the majority of the NVWA-IOD cases with risk categories A and B involve food laundering (14). Within these food laundering cases, there were no fraudulent changes made to the food product itself. The biological, chemical, or physical hazard arose in the production process. The cases first appeared as food safety events (accidental food contamination). During the investigation, it became clear that this involved the deliberate sale of these food products while hiding the food safety hazard from customers and consumers; in other words, the unsuitable or unsafe products were laundered into the food chain. Fraudulent food enhancement is less frequently associated with high-risk scores (A or B) in the NVWA-IOD cases. In only two cases, hazardous issues were reported due to product tampering: the addition of sibutramine in slimming tea and the addition of sulphite in minced meat.

Again, the risks found in the NVWA-IOD cases are more severe than those reported in the JRC reports, but still lower than the risks in the academic literature. NVWA-IOD prioritizes food fraud cases with (potential) negative effects on food safety, and these results suggest that the literature only focuses on the more severe cases.

### 3.4. Food Category

Figure 5 shows the number of reports per risk category (A–D) and food category (as defined in Section 2.4). The food category with the highest number of cases in the JRC data is the seafood category (177 cases), followed by ‘wine and alcoholic beverages’ and ‘meat and meat products’ (114 and 93 cases, respectively). These three groups form the top three in two other publications [28,29]; although ‘fruits and vegetables’ were less common there, ‘herbs and spices’ were more common. Everstine et al. [9] reported fraud with dairy products and fruit juices more often, but found fewer cases of ‘meat and meat products’. For Tähkäpää et al. [14], animal products were top of the list. Both the cases from the academic literature and from NVWA-IOD contained only a few examples of food categories outside the top 10 from the JRC, confirming that these ten categories likely form the most important group of commodities in terms of food fraud. The majority of the NVWA-IOD cases concern animal products (meat (products), aquatic foods (products), egg (products)). ‘Wine and alcoholic beverages’ and ‘fruit and vegetables’ are (almost) absent in the academic literature and NVWA-IOD cases, whereas egg (products) and ‘herbs and spices’ are relatively high in these two datasets. Given the relatively low number of cases, these differences are likely insignificant, and due to the general similarity, it confirms that the collected data does provide a sufficiently representative selection of actual cases of food fraud, where a deeper look into the cases is warranted.

### 3.5. In-Depth Observations on the JRC and Academic Literature Cases

It is interesting to investigate the common types of fraudulent actions per food category, for which the underlying data is available in Supplementary Table S3 (JRC cases) and Supplementary Table S4 (academic literature).

For ‘aquatic foods and products thereof’, many of the more serious cases describe the sale of expired foods or products unfit for consumption; sometimes even the qualification ‘rotten’ is used to describe the fraudulent products. Product treatment by the fraudsters to mask this issue is relatively rare, but document forgery to mask the issue is more common. Only a few cases describe treatments to restore or enhance the product’s colour. For this food category, the cases with little safety concern are cases of laundering products that are illegal due to exceeding fishing quota, and the lack of traceability of products that are mostly caught and distributed locally. In the cases reported in the academic literature, fish substitution is a recurring theme, where the danger stems from using species that naturally produce toxins (e.g., pufferfish), or where the substitute could trigger allergic reactions.

The ‘wine and alcoholic beverages’ category is probably relatively large due to the strong contribution of Southern European countries to the reports picked up by the JRC, which have a strong wine culture and probably also have substantial control measures in place. In terms of serious health risks, one case involved wine being used to disguise hard drug smuggling, a few involved fermentation with expired materials, and others involved the addition of non-food-grade alcohols. Most cases concerned some form of counterfeiting, inexpensive wine or spirits sold as more expensive ones, where the inexpensive substitute typically lacks traceability but is generally not more harmful than the genuine product. In addition, there are a number of cases of undeclared chaptalization (sugar addition) of wine. In the academic literature, a drug smuggling case and non-food-grade alcohol additions are reported.

‘Meat and meat products’ are in many ways similar to fishery products in the sense that most serious food safety risks originate from selling products unfit for human consumption. In some cases, it was even known to the suppliers that their products contained specific harmful microorganisms. Another recurring observation is that meat products are seized because slaughter took place illegally in facilities with sub-standard (hygienic) conditions. Apart from a single case of water addition, none of the JRC records indicate that meat products are treated with chemical agents. The milder forms of meat fraud, in terms of food safety risks, mainly concern products sold for a higher price, i.e., falsely claiming a certain more exclusive species or a production origin/protected designated origin (PDO) status. The cases reported in the academic literature are rather diverse in nature, where in addition to the problems encountered in the JRC cases, they also include suspicions of transmissible spongiform encephalopathy (TSE), and the addition of various organ tissues, bulking agents, colourants, and preservatives.

For the ‘fruit and vegetables’ category, there is one case where expired food was sold on the black market. The other risks observed mostly concerned foods lacking traceability in combination with processing/storage under poor hygienic conditions. Cases with lower food safety risks are mostly those where the origin is falsified or where conventional produce is sold as organic.

For ‘milk and milk products’, the more serious cases are again the sale of products known to be unfit for human consumption. In individual reported cases, milk was treated with caustic substances to mask its deteriorated state, or the product was known to be contaminated with Listeria or to contain medicine residues. Lack of traceability and poor hygienic conditions are reported for the other cases with more serious food safety risks. Counterfeiting-type cases typically fall in the low food safety risk category. In the cases reported in academic literature, the addition of various preservatives is most common.

In the other food categories, a wide range of different incidents are reported. The most recurring observation, certainly within the cases that pose a threat to consumer health, is the continued sale of foods that are expired, contaminated, or otherwise unsuitable for consumption.

### 3.6. In-Depth Observations on the NVWA-IOD Cases

**Figure 6:**
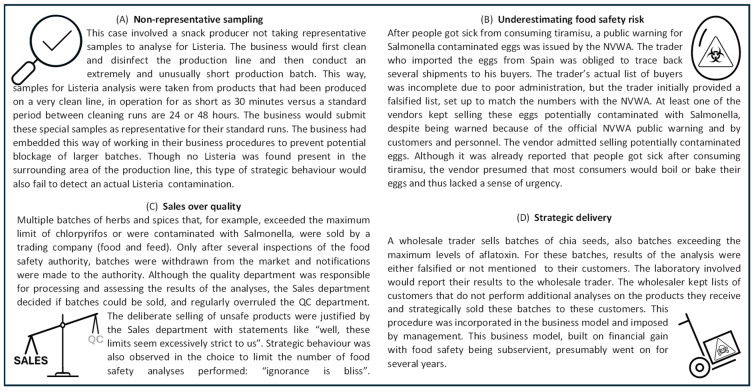
Illustrations describing case, severity, and motivations for a selection of NVWA-IOD cases: (**A**) Non-representative sampling, (**B**) Underestimating food safety risk, (**C**) Sales over quality, and (**D**) Strategic delivery.

The relative richness of the empirical material that is available on the 31 criminal investigations from the NVWA-IOD allowed a more in-depth analysis of the characteristics of the cases. This resulted in some additional observations about the link to food safety. Figure 6 contains several case descriptions that illustrate these observations.

Within the food fraud cases the NVWA-IOD conducted, three observations are noteworthy. The first observation is the strategic and fraudulent behaviour of food businesses with respect to the food safety monitoring processes. These should be in place to detect unsafe food products, allowing businesses to take appropriate measures to ensure these do not enter the market. The legislation defines acceptable or maximum levels for a range of substances to distinguish safe from unsafe. In several food laundering cases, food products were deliberately placed on the market while these levels were exceeded (for example, Figure 6C). This concerned a variety of food products—for example, black beans exceeding chlorpyrifos levels, yellow mustard contaminated with salmonella, rice, chia seeds and ground nuts with elevated aflatoxin levels and swordfish containing excessive methylmercury.

What stands out in these cases is the fraudulent behaviour within legitimate food businesses, resulting in the systematic undermining of food safety procedures. Results from monitoring processes were falsified or not mentioned on the analytical reports, batches of unsafe products were sold only to customers known not to conduct their own analysis, and no notifications were made to the food authority regarding unsafe products. By doing so, the food safety hazard was hidden from the customers as well as from the food authority. The businesses’ behaviour is aimed at minimizing the chance of detecting a food safety issue, which is also illustrated by the example in Figure 6A. Such fraudulent activity is likely to cause an underrepresentation of the actual food safety issues, especially if the fraudulent activity is structural and incorporated in the business model (Figure 6D). This behaviour creates a food safety issue by itself: a business that avoids the detection of food safety problems presumably does not take preventive or corrective measures to mitigate the food safety risk (Figure 6B), such as cleaning consignments, scrutinizing their suppliers, limiting purchases from high-risk countries, or recalling goods where a food safety issue might have been present.

To circumvent food safety procedures, food businesses sometimes use other businesses. The NVWA-IOD has investigated individuals or businesses engaging in activities that enable food fraud, defined as facilitative food fraud, such as servicing and logistics providers. In one of these cases, the investigation demonstrated that a laboratory deliberately did not report the positive lab results to the competent authority. The laboratory was employed by a poultry farm for the analysis of salmonella monitoring samples. To prevent an automatic notification from being sent to the competent authority, the laboratory manually checked a box within the database. By doing so, the food safety authority could not take veterinary measures. The poultry farm could thus continue to deliberately sell eggs to customers despite the salmonella contamination in one of the poultry houses, possibly for over a year. This led to several people becoming sick after eating homemade mayonnaise. Businesses such as laboratories are important pillars in the food safety system, and their complicit role raises the question about the actual scale of food safety risks, as their behaviour allows part of the food safety issues to stay under the radar rather than their risks being mitigated.

A second observation revolves around a number of similar cases, where the offenders are often not legitimate food businesses. These are connected specifically to the type of food laundering where previously recalled food products are reintroduced as food onto the market. The perpetrators are typically traders who are not registered with the food authorities, although this is legally required. This happened, for example, with batches of spice jars and chocolate spread, both possibly contaminated with glass particles. In both cases, the recalled batches were transferred to a waste processor for destruction but ended up being sold through internet advertisements and discount shops. In the case of the spice jars, the criminal investigation revealed that the waste collector reintroduced the spice jars onto the market by falsifying a declaration of destruction. This was discovered after a consumer reported an injury caused by using the spice jar to the food safety authority. As opposed to the first observation, this type of fraud seems to be more or less incidental, driven by opportunistic offenders with food targeted at the lower end of the market.

The final observation concerns cases where false statements about a food product are made to enhance its value. Though at first glance the associated food safety hazard seems low, it is the dishonesty that is inherent to fraudulent behaviour that can cause unexpected food safety risks. One such case of food fraud is where the geographic origin of oysters was fraudulently changed to a more expensive region: Irish oysters were sold as French oysters originating from the Charente. To make this seem plausible, many documents were forged, including quality control records, storage and shipping information, packaging, bills of lading, and invoices. The record tampering is thus more widespread than just mislabelling the final product. Such dishonesty can have far-reaching effects on food safety, as it corrupts food safety procedures throughout the supply chain. Other businesses in the supply chain that buy these products need correct information upon which to base their food safety measures, such as performing analyses at an appropriate, risk-based frequency. Other NVWA-IOD investigations with fraudulent statements about food products concerned butter (regular butter sold as meadow/pasture butter) and fraud resulting in conventionally produced meat sold with a private animal welfare label. Transport times of the cattle were fraudulently adjusted to meet the criteria of this label on paper.

## 4. Discussion

With respect to the primary aim of our study, the observations listed above give rise to some interesting insights into the relation between food fraud and food safety.

Firstly, the hazard type matters. The majority of hazards observed in the reported cases are of microbiological and chemical nature, where allergens and physical hazards are far fewer. To explain this, it can be assumed that fraudsters do not specifically aim to cause harm to consumers. Allergens are just a relatively small and quite specific proportion of available food (ingredients), so it seems likely that the incidence of allergens is lower. The same probably holds for physical threats; if there is little chance that whatever material the fraudster decides to use has a physical hazard to begin with, it is unlikely that fraud cases have such a hazard. In addition to the likelihood of being present, physical, allergenic, and some microbiological threats can cause acute effects, which might trigger the consumer to alert the authorities. If these reports can be properly followed up, it increases the chance of the threat being officially detected and reported as food fraud. Conversely, the effects of certain chemical threats can be far less acute, but could still be harmful over time, especially with repeated exposure. These threats are much less likely to be detected by consumers and rely only on controls, intelligence, or perhaps serendipity to be detected and identified as food fraud. The extent to which this leads to fewer reports in the databases is unclear, but it is plausible that non-acute (chemical) hazards are (even) more underestimated than those with acute effects, particularly because long-term health effects make it difficult to identify the exact cause and even harder to link them to food fraud. This difference in ‘detectability’ could also explain why products with increased risk of foodborne illnesses, like aquatic species, meat, milk, fruits and vegetables, and eggs are not only frequently reported, but also tend to represent higher risk categories. Fraudulent activities with these types of products, for example, employing illegal slaughter without veterinary inspections on the (health of the) animal before and after it is slaughtered, or tampering with production dates, can elevate this risk. Although all hazard types are a risk for food safety, the majority of more serious risks are found in the microbiological and chemical categories, making prevention efforts in those areas probably the most effective choice.

The types of food fraud studied by scientists involved in developing analytical methods to detect food fraud are most commonly product enhancement, the type of fraud where a food product is physically ‘upgraded’, commonly referred to as adulteration or substitution. This has been described as the ‘original’ form of food fraud [30]. Based on our findings, this type of food fraud is not the most frequent problem, nor the type that is associated with the most severe food safety issues. Instead, food laundering seems to be the predominant form of fraud, which also carries the larger food safety threat. This realization may be used to develop alternative strategies or additional analytical tools to detect more fraud cases analytically.

The food types most vulnerable to fraud, and also leading to most serious food safety issues, are perishable foods. Frequently, microbial issues potentially leading to foodborne illnesses are responsible for the risk, but fraudulent actions intended to prevent the development of foodborne illnesses also happen, as well as fraudulent actions to prevent the detection of any safety concerns. Surprisingly, given their large and increasing share of the current food market, the number of fraudulent cases concerning mixed and prepared foods and complete meals is relatively low, and the risk for the cases that are found is quite low. It seems logical that the greater the number of raw materials mixed, the higher the likelihood that at least one is affected by food fraud, but our data does not indicate whether it is the chance or the detectability of fraud that decreases for this product group.

What stands out in the results is that safeguarding food safety is not as evident as may be generally assumed. Food safety incidents are still generally considered an accidental event, but this study demonstrates how food fraud is largely aimed at consciously ignoring food safety regulations and risks, either by deliberately not implementing appropriate measures to detect food safety issues, or by avoiding the appropriate follow-up actions. In addition, contaminated or depreciated foodstuffs, even if discarded by one business, attract other businesses that try to benefit from them and reintroduce them to the market. Consciously ignoring food safety is the real threat of fraudulent behaviour by food businesses, and contrasts sharply with the European General Food Law that aims to bring a food safety culture to the food industry. Moreover, the intent to hide issues also means that this fraudulent and possibly unsafe conduct is likely to remain undiscovered to a large extent, and further complicates the traceability and safety assurance of foods produced by these actors.

A secondary aim of this study was to test the conceptualization of food fraud as suggested by Gussow & Mariet [12]. Applying these concepts to this dataset worked well methodologically. After discussing the concepts and expanding them with subcategories that we thought relevant from a food safety perspective, we were able to easily assign the different concepts to the data entries. The distinction between illegal or unfit food and legal food as an initial step to identify the type of food fraud proved helpful. A few types of food fraud were then discussed together, such as how to classify illegally produced foods—for example, those produced under unhygienic circumstances (food laundering of unknown food grade). In some cases, both product and record tampering occurred. These cases were classified as product tampering, assuming that record tampering is likely to also be present. The point, however, is to investigate the occurrence of actual product tampering and the associated risks and product categories that made this the most important to register. These insights enriched the picture of types of food fraud and were used to clarify our view on food fraud, as visualized in the model in Figure 2.

In terms of the results, using these food fraud categories proved to be insightful. It clearly distinguishes fraudulent food enhancement, through either product or record tampering, on the one hand, which seems to be the dominant view in food fraud definitions [31], from other illegal activities with regard to food, i.e., food laundering. The JRC dataset, which is a fairly broad empirical dataset of food fraud incidents that have come to light, shows that food laundering is found to occur more often and seems to be a larger food safety threat than food enhancement (Section 3.3). It is thus an important category that warrants further exploration as has been discussed above.

Furthermore, this classification allows us to see and understand the different focus of the three datasets. Applying this definition to the JRC cases reinforces the observation by Gussow & Mariët [12] that food laundering is an important type of food fraud that occurs throughout the EU, but one that academic literature rarely addresses in terms of prevention and detection. Notably, facilitative food fraud was hardly found outside of the NVWA-IOD dataset. The associated harms can, however, be considerable, as in cases where laboratories facilitate the sale of unsafe foods. This raises the question of whether authorities and academic researchers are sufficiently aware of this type of food fraudster. In summary, this conceptualization provided analytically useful insights and revealed that food laundering is a blind spot in food fraud that is largely overlooked in the academic research on prevention and detection strategies, among which is analytical method development.

## 5. Conclusions and Recommendations

Based on the data found, with its limitations in terms of coverage and completeness, we conclude that roughly one third of documented food fraud cases are regarded as posing a considerable food safety risk. While food safety incidents are still generally considered accidental events, this study shows that a large proportion of food fraud involves the deliberate disregard of food safety regulations and associated risks. Based on the cases we studied, food laundering seems to be the predominant form of fraud, which also carries the larger food safety threat. Product enhancement (adulteration) has received much attention in the analytical community, but this type of food fraud is unlikely to be the most frequent or pose the highest risk. Although all hazard types are a risk for food safety, the majority of more serious risks are found in the microbiological and chemical categories, suggesting that prevention efforts targeting these categories are likely the most effective. In our data, the food types most vulnerable to fraud, and also leading to most serious food safety issues, are perishable foods. Finally, the conceptualization of food fraud as suggested by Gussow & Mariet [12], with expanding subcategories, proved useful for analysing food safety in relation to food fraud and revealed food laundering as a critical blind spot.

Food fraud is not only undesirable but inherently unsafe, and improved options for both prevention and detection need to be developed. Building on the conclusions above, the emphasis on the food safety effects of food fraud in this paper could be used to guide future research, regulators and industries, both for prevention and detection. Since the technology for analytical detection of actual hazards is already established, these techniques could complement the predominantly multivariate methods that are currently being developed. If adapted for broader, non-targeted application, some of these techniques could evolve into dual-use tools for both hazard and food fraud detection. Regulators need to be more aware of the reluctance of industries to depreciate foodstuffs and their fraudulent behaviour to disguise food safety issues. Industries could review their fraud vulnerability assessments to incorporate the insights that food laundering results in unauthorised batches being (re)introduced in the food supply chain.

Another clear direction for future research is developing guidance on where to look and which hazards to target. Not unlike other safety mitigation, risk estimations are needed to prioritise detection and prevention efforts. Some patterns found in this study could be a starting point to develop further, keeping in mind that patterns found may be based on the previously detected fraud cases only. For some, detecting and preventing hazardous cases might be a priority over less harmful forms of food fraud. Effective safety mitigation not only requires technical knowledge on how possible food hazards might exist and develop, but also insight into the behaviour of many actors in the food chain —understanding why, how, and when decisions are made that (may) lead to unsafe food.

## Data Availability

All data are available in the manuscript and Supplementary Files.

## Supplementary Materials

The following supporting information can be downloaded at: https://www.mdpi.com/article/10.3390/foods14244328/s1, Supplementary Figures S1 and S2: Graphical representations of the number of entries per year; Supplementary Table S1: Search queries; Supplementary Table S2: Eligibility criteria; Supplementary Table S3: List of JRC cases considered in this study; Supplementary Table S4: List of literature cases considered in this study; Supplementary Table S5: List of NVWA-IOD cases considered in this study; Supplementary References [7,24,25,26,27,32,33,34,35,36,37,38,39,40,41,42,43,44,45,46,47,48,49,50,51,52,53,54,55,56,57,58,59,60,61,62,63,64,65,66,67,68,69,70,71,72,73,74]: References for literature quoted in Supplementary Table S4.

## Abbreviations

The following abbreviations are used in this manuscript:

JRC	Joint Research Centre
GFSI	Global Food Safety Initiative
ACN	Alert and Cooperation Network
CEN	European Committee for Standardization
EU	European Union
FSTA	Food Science and Technology Abstracts
NVWA	Netherlands Food and Consumer Product Safety Authority
NVWA-IOD	Intelligence and Investigation Service of the Netherlands Food and Consumer Product Safety Authority
Al.	Allergenic
Bi.	Biological
Ch.	Chemical
Ph.	Physical
Laun_non	Food laundering, non-food grade
Laun_unk	Food laundering, unknown grade
Laun_food	Food laundering, food grade
Enh_prod	Fraudulent food enhancement, product tampering
Enh_rec	Fraudulent food enhancement, record tampering
Fac_prod	Facilitation, product tampering
Fac_rec	Facilitation, record tampering
Fac_laun	Facilitation, food laundering
ASAE	Economic and Food Safety Authority
NAS	Anti-adulteration and healthcare unit of the Carabinieri
TSE	Transmissible Spongiform Encephalopathy
