# Supplementary material for: An Analysis of the Severity of Food Safety Hazards in EU Food Fraud Cases
Source: Foods. 2025 Dec 16;14(24):4328. doi: 10.3390/foods14244328 (PMC12732713; doi:10.3390/foods14244328)
Supplement: Supplementary file 1 [file foods-14-04328-s001.zip › foods-3990759-supplementary.pdf]

Supplementary material belonging to manuscript entitled “An analysis of the severity of food safety hazards in EU food fraud cases”

Brief content description:

• Supplementary Figures S1 – S2	Graphical representations of the number of entries per year.
• Supplementary Table S1	Search queries
• Supplementary Table S2	Eligibility criteria
• Supplementary Table S3	List of JRC cases considered in this study
• Supplementary Table S4	List of literature cases considered in this study
• Supplementary Table S5	List of NVWA-IOD cases considered in this study

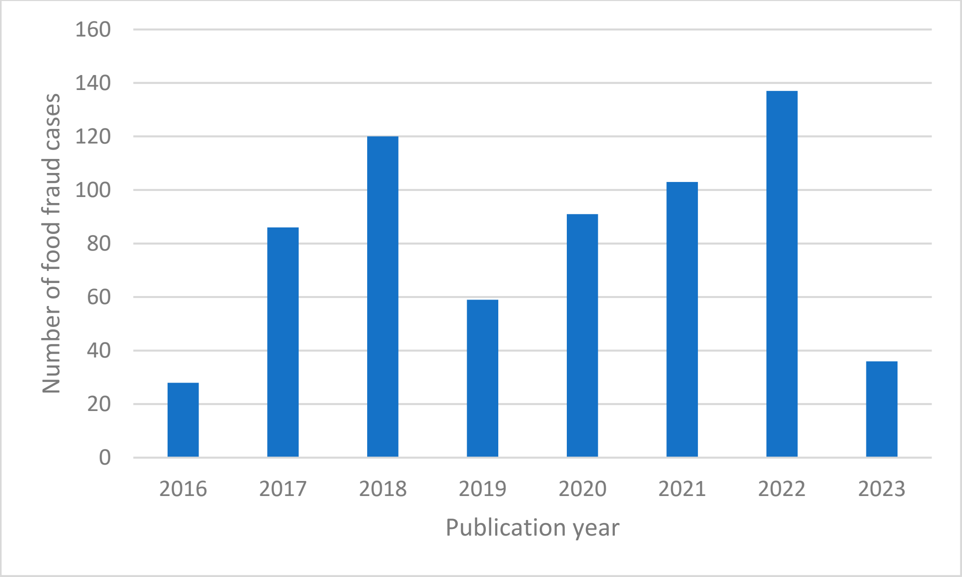

Supplementary Figure S1. Number of included food fraud cases sourced from the JRC monthly summaries published between 2016 and 2023 (n=659). Only four months of summaries were available to review in 2016 (September-December) and only three months were available in 2023 (January – March). Where multiple food products were mentioned in one news item on food fraud, these were counted as separate cases.

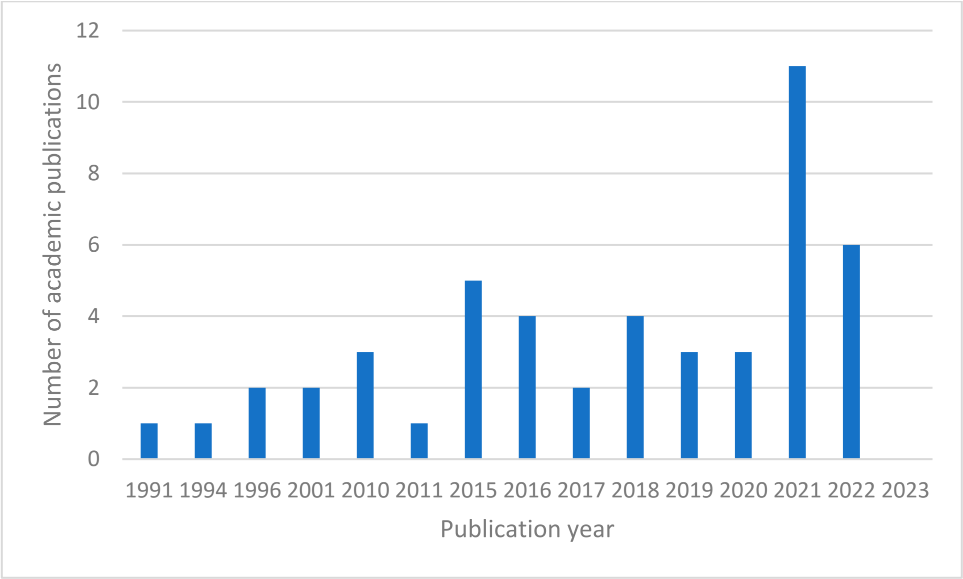

Supplementary Figure S2. Number of included academic publications sourced from literature databases published between 1991 and 2023 (n=48). No relevant academic publications were published in 2023 at the time of this review.

Supplementary Table S1. Search string run on each database, the date the search was run, and the subsequent number of records obtained.

Where search was run/database	Search string	Search date (day/month/year)	Number of records
**Food Science and Technology Abstracts (FSTA)**	1. foods/<br>2. food supplements/ or supplements/<br>3. 1 or 2<br>4. food fraud/ or adulteration/ or authenticity/ or fraud/<br>5. food safety/ or food safety additives/ or food safety beverages/ or food safety plant foods/ or european food safety authority/ or hazards/ or hazards analysis/ or health/ or health hazards/ or poisoning/ or product recalls/ or public health/ or risks assessment/ or risks management/ or toxicology/ or toxins/<br>6. european union/ or austria/ or belgium/ or cyprus/ or czech republic/ or denmark/ or estonia/ or finland/ or france/ or germany/ or greece/ or hungary/ or italy/ or latvia/ or lithuania/ or luxembourg/ or malta/ or netherlands/ or poland/ or portugal/ or "republic of ireland"/ or slovakia/ or slovenia/ or spain/ or sweden/ or united kingdom/ or europe/ or european community/<br>7. 3 and 4 and 5 and 6	11/05/2023	22
**PubMed**	((((food) OR (food supplements)) AND (fraud OR adulteration OR authenticity)) AND (safety OR hazards OR (health hazards) OR health OR poisoning OR (product recalls) OR risk OR toxicology OR toxins OR rasff)) AND ((European Union) OR EU OR Europe)	11/05/2023	333
**Web of Science Core Collection**	(((ALL=(food OR 'food products' OR 'food supplements')) AND ALL=(fraud OR frauds OR adulteration OR authenticity)) AND ALL=(safety OR hazards OR hazard OR health OR poisoning OR ‘product recalls’ OR risk OR toxicology OR toxins OR RASFF)) AND ALL=(‘European Union’ OR European OR EU OR Europe)	11/05/2023	605
**Scopus**	food OR 'food AND products' OR 'food AND supplements' AND fraud OR adulteration OR authenticity AND safety OR hazard OR hazards OR health OR poisoning OR risk OR toxicology AND 'european AND union' OR eu OR europe	11/05/2023	1791

Supplementary Table S2. Eligibility criteria used for deliberately including or excluding food fraud cases and academic publications from the review.

**Inclusion criteria**
Revolve around food fraud
Human safety risk
The safety risk must be linked to food fraud
Within or affect the European Union
Any food product/ food supplements [a]
Both qualitative and quantitative studies
Published research articles, reviews, journals, books, conference proceedings, theses, reports, fraud databases
**Exclusion criteria**
Not concerning food
Not concerning food fraud
Concerning food fraud but with no link to food safety
Only considering food safety
Does not affect the European Union/only concerns in a country outside the European Union
Animal feed
Animal safety risks
Food defence
Herbal medicines/food supplements [a]
States that there is a link between food fraud and food safety but does not elaborate or explain further [b]

[a] Food supplements was moved from the inclusion criteria to the exclusion criteria during the screening process. [b] This criterion was put in place during full-text screening.

Supplementary Table S3. Qualitative summary table of food fraud cases concerning the European Union with a food safety risk taken from the JRC monthly summaries between September 2016 up to and including March 2023.

Period	Issue	Product category	Food fraud category	Food fraud subcategory	Hazard	Severity category
Sep-16	Seafood without traceability	Aquatic foods and products thereof	Food laundering	Food grade	Biological/chemical	D
Sep-16	Unlabelled wine lacking traceability and documentation, and poor hygienic conditions	Wine and alcoholic beverages	Food laundering	Unknown food grade	Biological	C
Sep-16	Bluefin tuna and swordfish without label of origin	Aquatic foods and products thereof	Food laundering	Unknown food grade	Biological/chemical	C
Sep-16	"anonymous" fish being transported in a freezer truck	Aquatic foods and products thereof	Food laundering	Food grade	None	D
Sep-16	Seizure of fish related to mislabelling of seafood products	Aquatic foods and products thereof	Food laundering	Unknown food grade	Biological	D
Sep-16	Concentrated grape must with false documentation	Wine and alcoholic beverages	Fraudulent food enhancement	Record tampering	None	D
Oct-16	Seizure of fish and seafood	Aquatic foods and products thereof	Food laundering	Unknown food grade	Biological/chemical	D
Oct-16	Table grape must lacking necessary documentation	Wine and alcoholic beverages	Food laundering	Unknown food grade	None	D
Oct-16	Grape must with falsified documentation	Wine and alcoholic beverages	Food laundering	Unknown food grade	None	D
Oct-16	Watered-down Italian wine: dispute over the chemical and statistical analysis	Wine and alcoholic beverages	Fraudulent food enhancement	Product tampering	None	D
Nov-16	Importation of unlabelled/mislabelled Asian fish and pacific clams	Aquatic foods and products thereof	Fraudulent food enhancement	Record tampering	Biological/chemical	C
Nov-16	Untraceable eggs some pallets with altered visual and organoleptic characteristics	Eggs and egg products	Food laundering	Non-food grade	Biological	C
Nov-16	Olive oil diluted with other vegetable oils	Fats and oils	Fraudulent food enhancement	Product tampering	Allergenic	C
Nov-16	Operation Sapori d'autunno: Seizure of variety of Autumn seasonal products with labelling irregularities	Meat and meat products	Fraudulent food enhancement	Record tampering	Biological/chemical	D
Nov-16	Operation Sapori d'autunno: Seizure of variety of Autumn seasonal products with labelling irregularities	Milk and milk products	Fraudulent food enhancement	Record tampering	Biological/chemical	D
Nov-16	Operation Sapori d'autunno: Seizure of variety of Autumn seasonal products with labelling irregularities	Other food/mixed	Fraudulent food enhancement	Record tampering	Biological/chemical	D
Nov-16	Honey, jam and olive oil labelled as made in Ischia (island west of Naples) originated from continental Italy	Honey and royal jelly	Fraudulent food enhancement	Record tampering	None	D
Nov-16	Honey, jam and olive oil labelled as made in Ischia (island west of Naples) originated from continental Italy	Fats and oils	Fraudulent food enhancement	Record tampering	None	D

Period	Issue	Product category	Food fraud category	Food fraud subcategory	Hazard	Severity category
Dec-16	Mislabelling and poor hygienic conditions	Aquatic foods and products thereof	Food laundering	Non-food grade	Biological	D
Dec-16	Lack of labelling and thawed product sold as fresh	Aquatic foods and products thereof	Fraudulent food enhancement	Record tampering	Biological	C
Dec-16	Untraceable fish and seafood	Aquatic foods and products thereof	Food laundering	Unknown food grade	Biological/chemical	C
Dec-16	Unlabelled fish	Aquatic foods and products thereof	Food laundering	Unknown food grade	Biological/chemical	C
Dec-16	Stolen animals introduced illegally in the food chain with fake documentation and illegal slaughtering	Meat and meat products	Food laundering	Non-food grade	Biological/chemical	C
Dec-16	Lack of labelling and origin masking of many food products in a Chinese supermarket in Italy	Cereals and bakery products	Food laundering	Unknown food grade	Allergenic	D
Dec-16	Lack of labelling and origin masking of many food products in a Chinese supermarket in Italy	Fruits and vegetables	Food laundering	Unknown food grade	Allergenic	D
Dec-16	Lack of labelling and origin masking of many food products in a Chinese supermarket in Italy	Non-alcoholic beverages	Food laundering	Unknown food grade	Allergenic	D
Dec-16	Lack of labelling and origin masking of many food products in a Chinese supermarket in Italy	Other food/mixed	Food laundering	Unknown food grade	Allergenic	D
Dec-16	Production of adulterated wine infringing several rules such as the addition of alcohol, reproduction of counterfeit high-quality wine labels, and illegal attribution of official Protected Designation of Origin seals to the bottles	Wine and alcoholic beverages	Fraudulent food enhancement	Record tampering	Chemical	C
Jan-17	Mislabelling of fish products and lack of traceability	Aquatic foods and products thereof	Food laundering	Unknown food grade	Biological/chemical	D
Jan-17	Mislabelling of fish products and lack of traceability	Aquatic foods and products thereof	Food laundering	Unknown food grade	Biological/chemical	D
Jan-17	Untraceable fish	Aquatic foods and products thereof	Food laundering	Unknown food grade	Biological/chemical	D
Jan-17	Processed vegetables in oil lacking any labelling and not fit for consumption	Fruits and vegetables	Food laundering	Non-food grade	Biological/physical	C
Jan-17	Counterfeit white wine	Wine and alcoholic beverages	Fraudulent food enhancement	Record tampering	None	D
Jan-17	Counterfeit wine of exclusive high quality French brand	Wine and alcoholic beverages	Fraudulent food enhancement	Record tampering	None	D
Feb-17	Octopus with no traceability records	Aquatic foods and products thereof	Food laundering	Unknown food grade	Biological/chemical	D
Feb-17	Olive oil fraudulently diluted with other vegetable oils	Fats and oils	Fraudulent food enhancement	Product tampering	Allergenic	D
Feb-17	Exportation of cheap olive pomace oil relabelled as extra virgin	Fats and oils	Fraudulent food enhancement	Record tampering	None	C

Period	Issue	Product category	Food fraud category	Food fraud subcategory	Hazard	Severity category
Feb-17	Sicilian tomatoes actually from Albania	Fruits and vegetables	Fraudulent food enhancement	Record tampering	None	D
Feb-17	PDO protected ham was produced from inappropriate animals, documents forged to conceal this.	Meat and meat products	Fraudulent food enhancement	Product tampering	Chemical	C
Feb-17	Spoiled milk treated with caustic soda and cow's milk instead of buffalo's milk used to make PDO mozzarella di bufala	Milk and milk products	Fraudulent food enhancement	Record tampering	Allergenic/biological/chemical	B
Mar-17	Seizure of fish for lack of or incomplete traceability labelling	Aquatic foods and products thereof	Food laundering	Unknown food grade	Biological/chemical	D
Mar-17	Seizure of fish and seafood lacking traceability	Aquatic foods and products thereof	Food laundering	Unknown food grade	Biological/chemical	D
Mar-17	lack of traceability and proper labelling of fish products	Aquatic foods and products thereof	Food laundering	Unknown food grade	None	D
Mar-17	Eggs with missing or incomplete declaration of origin and other labelling information	Eggs and egg products	Food laundering	Unknown food grade	Biological	D
Mar-17	Unlabelled processed food found unlabelled in Chinese food shop	Other food/mixed	Food laundering	Non-food grade	Allergenic/biological	C
Mar-17	Seizure of wine and spirits with forged labels and fake marks	Wine and alcoholic beverages	Fraudulent food enhancement	Record tampering	None	D
Apr-17	wrong or absence of labelling	Eggs and egg products	Food laundering	Unknown food grade	Biological	D
Apr-17	Poultry meat coming from Brazil contaminated with salmonella	Meat and meat products	Food laundering	Non-food grade	Biological	B
Apr-17	untraceable frozen goats milk	Milk and milk products	Food laundering	Unknown food grade	Biological/chemical	D
Apr-17	Comprehensive food sector operation by Europol and Interpol (seizure of food and beverages) alcohol, mineral water, seasoning cubes, seafood, caviar, olive oil	Wine and alcoholic beverages	Fraudulent food enhancement	Record tampering	Allergenic/biological/chemical	C
Apr-17	Comprehensive food sector operation by Europol and Interpol (seizure of food and beverages) alcohol, mineral water, seasoning cubes, seafood, caviar, olive oil	Non-alcoholic beverages	Fraudulent food enhancement	Record tampering	Allergenic/biological/chemical	C
Apr-17	Comprehensive food sector operation by Europol and Interpol (seizure of food and beverages) alcohol, mineral water, seasoning cubes, seafood, caviar, olive oil	Soups, broths, sauces and condiments	Fraudulent food enhancement	Record tampering	Allergenic/biological/chemical	C
Apr-17	Comprehensive food sector operation by Europol and Interpol (seizure of food and beverages) alcohol, mineral water, seasoning cubes, seafood, caviar, olive oil	Aquatic foods and products thereof	Fraudulent food enhancement	Record tampering	Allergenic/biological/chemical	C
Apr-17	Comprehensive food sector operation by Europol and Interpol (seizure of food and beverages) alcohol, mineral water, seasoning cubes, seafood, caviar, olive oil	Fats and oils	Fraudulent food enhancement	Record tampering	Allergenic/biological/chemical	C

Period	Issue	Product category	Food fraud category	Food fraud subcategory	Hazard	Severity category
May-17	Lack of mandatory labelling fo fish and cases of species substitution	Aquatic foods and products thereof	Fraudulent food enhancement	Product tampering	Biological/chemical	D
May-17	Restaurant suspected of selling low grade fish species instead of the higher quality fishes	Aquatic foods and products thereof	Fraudulent food enhancement	Product tampering	Biological/chemical	D
May-17	Pig meat, soy and bread added to beef burgers and meatballs	Meat and meat products	Fraudulent food enhancement	Product tampering	Allergenic	C
May-17	Sausages made with pig meat instead of the advertised wild boar and deer meat	Meat and meat products	Fraudulent food enhancement	Product tampering	None	D
May-17	Historic trademark from San Gimignano was counterfeited with the help of fake labels	Wine and alcoholic beverages	Fraudulent food enhancement	Product tampering	None	D
Jun-17	Mislabelled fish lacking origin and traceability documentation	Aquatic foods and products thereof	Food laundering	Non-food grade	Allergenic/biological/chemical	D
Jun-17	Frozen fish lacking mandatory labelling	Aquatic foods and products thereof	Food laundering	Unknown food grade	Biological/chemical	D
Jun-17	Untraceable fish	Aquatic foods and products thereof	Food laundering	Unknown food grade	Biological/chemical	D
Jun-17	Lack of origin labelling	Aquatic foods and products thereof	Food laundering	Unknown food grade	Biological/chemical	D
Jun-17	Illegally caught octopus	Aquatic foods and products thereof	Food laundering	Unknown food grade	Biological/chemical	D
Jun-17	Meat seized, due to lack of licensing, lack of Veterinary Control Number and lack of traceability	Meat and meat products	Fraudulent food enhancement	Product tampering	Biological/chemical	B
Jun-17	Cheese in the process of maturation lacking any labelling making it totally untraceable	Milk and milk products	Food laundering	Unknown food grade	Biological/chemical	C
Jun-17	Illegal use of a reputed brand of Portuguese wine	Wine and alcoholic beverages	Food laundering	Unknown food grade	None	D
Jul-17	Fipronil (banned insecticide) ended up in eggs	Eggs and egg products	Facilitation	Facilitates product tampering	Chemical	B
Jul/Aug-17	Expired and/or unlabelled products put on sale	Aquatic foods and products thereof	Food laundering	Non-food grade	Biological/chemical	C
Jul/Aug-17	Lack of labelling and traceability of fish and seafood.	Aquatic foods and products thereof	Food laundering	Unknown food grade	Biological/chemical	D
Jul/Aug-17	Several traceability problems in an egg production company	Eggs and egg products	Food laundering	Unknown food grade	Biological	D
Jul/Aug-17	Dried zucchini labelled as organic but not accompanied with the necessary certificates	Fruits and vegetables	Fraudulent food enhancement	Record tampering	Chemical	D
Jul/Aug-17	Distribution of horsemeat unfit for human consumption	Meat and meat products	Food laundering	Non-food grade	Chemical	B
Jul/Aug-17	Selling frozen food in a restaurant without properly mentioning it on the menu	Prepared dishes and snacks	Fraudulent food enhancement	Record tampering	None	D

Period	Issue	Product category	Food fraud category	Food fraud subcategory	Hazard	Severity category
Jul/Aug-17	Imported wine not complying with mandatory labelling requirements	Wine and alcoholic beverages	Fraudulent food enhancement	Record tampering	None	D
Jul/Aug-17	Illegal use of an image of a well-known and respected wine brand on the national market	Wine and alcoholic beverages	Fraudulent food enhancement	Record tampering	None	D
Jul/Aug-17	Selling cheap wine under fake denominations	Wine and alcoholic beverages	Fraudulent food enhancement	Record tampering	None	D
Sep-17	Treating thawed red tuna with beet and vegetables broth to rejuvenate the fish	Aquatic foods and products thereof	Fraudulent food enhancement	Product tampering	Biological/chemical	A
Sep-17	Unlabelled fish products hidden among regular products	Aquatic foods and products thereof	Food laundering	Unknown food grade	Biological/chemical	D
Sep-17	Guatemalan coffee mixed with other coffee from Vietnam and Uganda and packaging bearded a counterfeited trademark	Cocoa and cocoa preparations, coffee and tea	Fraudulent food enhancement	Product tampering	None	D
Sep-17	Fontina produced in unregistered places, labelled with forged information and entered fraudulently into the production chain	Milk and milk products	Food laundering	Unknown food grade	Biological	D
Sep-17	Dairy farms not accredited for the production of such cheese and re-used old labels to pass the product as authentic	Milk and milk products	Food laundering	Unknown food grade	Biological/chemical	D
Sep-17	Chaptalization of wine	Wine and alcoholic beverages	Fraudulent food enhancement	Product tampering	None	D
Sep-17	Falsely labelling a table wine with a PGI label	Wine and alcoholic beverages	Fraudulent food enhancement	Product tampering	None	D
Oct-17	Unlabelled fish and molluscs	Aquatic foods and products thereof	Food laundering	Unknown food grade	Biological/chemical	D
Oct-17	Transport of untraceable fish	Aquatic foods and products thereof	Food laundering	Unknown food grade	Biological/chemical	D
Oct-17	Pure oregano cut with ground dry leaves from olive or myrtle trees	Herbs and spices	Fraudulent food enhancement	Product tampering	None	C
Oct-17	Sector of ice cream and granité during the summer months showed several violations of labelling rules	Ices and desserts	Fraudulent food enhancement	Record tampering	Biological	D
Oct-17	Several serious labelling irregularities for many products in restaurants: fish, meat, ice cream, dairy products	Aquatic foods and products thereof	Food laundering	Unknown food grade	Biological/chemical	C
Oct-17	Several serious labelling irregularities for many products in restaurants: fish, meat, ice cream, dairy products	Meat and meat products	Food laundering	Unknown food grade	Biological/chemical	C
Oct-17	Several serious labelling irregularities for many products in restaurants: fish, meat, ice cream, dairy products	Milk and milk products	Food laundering	Unknown food grade	Biological/chemical	C
Oct-17	Several cases of grape theft in France following poor growing season	Wine and alcoholic beverages	Food laundering	Food grade	None	D
Oct-17	Wine bottles bearing counterfeit labels and containing wine that did not comply with the PDO making process	Wine and alcoholic beverages	Fraudulent food enhancement	Product tampering	None	D

Period	Issue	Product category	Food fraud category	Food fraud subcategory	Hazard	Severity category
Nov-17	Fish without traceability	Aquatic foods and products thereof	Food laundering	Unknown food grade	Biological/chemical	D
Nov-17	Non-compliance with current regulations on traceability	Aquatic foods and products thereof	Food laundering	Unknown food grade	Biological/chemical	D
Nov-17	Fish without traceability	Aquatic foods and products thereof	Food laundering	Unknown food grade	Biological/chemical	D
Nov-17	Transport, possession and marketing of undersized fish	Aquatic foods and products thereof	Food laundering	Food grade	Biological/chemical	D
Nov-17	Fishing, possession and sale of albacore tuna, undersized fish products and absence of mandatory information	Aquatic foods and products thereof	Food laundering	Unknown food grade	Biological/chemical	D
Nov-17	Criminal offences related to fishing	Aquatic foods and products thereof	Food laundering	Unknown food grade	Biological/chemical	D
Nov-17	Lack of commercial documents certifying origin and expected traceability	Aquatic foods and products thereof	Food laundering	Unknown food grade	None	D
Nov-17	Illegally fish bluefin tuna with no traceability or labels	Aquatic foods and products thereof	Food laundering	Unknown food grade	None	D
Nov-17	Eggs seized for lack of traceability	Eggs and egg products	Food laundering	Unknown food grade	Biological	D
Nov-17	Cheap olive oil was actually dyed sunflower oil	Fats and oils	Fraudulent food enhancement	Product tampering	Allergenic/chemical	C
Nov-17	Theft of olives directly from the field as well as traceability problems	Fats and oils	Food laundering	Unknown food grade	Chemical	D
Nov-17	Products cultivated by conventional methods using pesticides labelled as organic	Fruits and vegetables	Fraudulent food enhancement	Product tampering	Chemical	D
Nov-17	PDO fraud on honey	Honey and royal jelly	Fraudulent food enhancement	Product tampering	None	D
Nov-17	Flower honey marketed as acacia	Honey and royal jelly	Fraudulent food enhancement	Product tampering	None	D
Nov-17	Untraceable meat seized at a big distributor + poor hygiene	Meat and meat products	Food laundering	Unknown food grade	Biological	C
Nov-17	Unlabelled and untraceable olive oil and unlabelled red wine stored in unregistered barrels	Fats and oils	Food laundering	Unknown food grade	Chemical	D
Nov-17	Unlabelled and untraceable olive oil and unlabelled red wine stored in unregistered barrels	Wine and alcoholic beverages	Food laundering	Unknown food grade	Chemical	D
Nov-17	Fake Chianti, the reputed Protected Denomination of Origin (PDO) wine from Tuscany	Wine and alcoholic beverages	Fraudulent food enhancement	Product tampering	None	D
Dec-17	Traceability of fish	Aquatic foods and products thereof	Food laundering	Unknown food grade	Biological/chemical	D
Dec-17	Infractions concerning mislabelling	Aquatic foods and products thereof	Food laundering	Unknown food grade	Biological/chemical	D

Period	Issue	Product category	Food fraud category	Food fraud subcategory	Hazard	Severity category
Dec-17	Irregularities concerning labelling and traceability	Aquatic foods and products thereof	Food laundering	Unknown food grade	None	D
Jan-18	Untraceable fish	Aquatic foods and products thereof	Food laundering	Unknown food grade	Biological/chemical	D
Jan-18	Counterfeiting San Daniele ham, protected by a PDO	Meat and meat products	Fraudulent food enhancement	Product tampering	None	D
Jan-18	Lack of traceability: confectionary, eggs	Cereals and bakery products	Food laundering	Unknown food grade	Biological	D
Jan-18	Lack of traceability: confectionary, eggs	Eggs and egg products	Food laundering	Unknown food grade	Biological	D
Jan-18	Substandard wine with added alcohol sold under reputed denominations	Wine and alcoholic beverages	Fraudulent food enhancement	Product tampering	None	C
Jan-18	Commercializing fake Franciacorta wine, a reputed PDO sparkling wine	Wine and alcoholic beverages	Fraudulent food enhancement	Product tampering	None	D
Feb-18	Untraceable frozen fish	Aquatic foods and products thereof	Food laundering	Unknown food grade	Biological/chemical	D
Feb-18	Molluscs with no traceability or documentation of origin	Aquatic foods and products thereof	Food laundering	Unknown food grade	Biological/chemical	D
Feb-18	Lack of traceability in the fish sector	Aquatic foods and products thereof	Food laundering	Unknown food grade	Biological/chemical	D
Feb-18	Fish served in restaurants often differs from the one stated on the menu	Aquatic foods and products thereof	Fraudulent food enhancement	Product tampering	None	D
Feb-18	Fake organic coffee	Cocoa and cocoa preparations, coffee and tea	Fraudulent food enhancement	Product tampering	None	D
Feb-18	Fruits with no labelling of origin	Fruits and vegetables	Food laundering	Unknown food grade	Biological/chemical	D
Feb-18	Substandard saffron labelled as highest quality	Herbs and spices	Fraudulent food enhancement	Record tampering	None	D
Feb-18	Fake Iberico hams, a product with a PDO	Meat and meat products	Fraudulent food enhancement	Product tampering	None	D
Feb-18	Untraceable food found during a wide control operation: fish, cheese, bakery products, fresh pasta	Aquatic foods and products thereof	Food laundering	Unknown food grade	Biological/chemical	D
Feb-18	Untraceable food found during a wide control operation: fish, cheese, bakery products, fresh pasta	Milk and milk products	Food laundering	Unknown food grade	Biological/chemical	D
Feb-18	Untraceable food found during a wide control operation: fish, cheese, bakery products, fresh pasta	Cereals and bakery products	Food laundering	Unknown food grade	Biological/chemical	D
Feb-18	Untraceable food in ethnic restaurants	Prepared dishes and snacks	Food laundering	Unknown food grade	Biological/chemical	D

Period	Issue	Product category	Food fraud category	Food fraud subcategory	Hazard	Severity category
Feb-18	Counterfeit of the traditional Aceto Balsamico di Modena	Soups, broths, sauces and condiments	Fraudulent food enhancement	Product tampering	None	D
Mar-18	Portuguese clam sold as Galician	Aquatic foods and products thereof	Fraudulent food enhancement	Product tampering	Biological/chemical	D
Mar-18	Blending olive oil with other oils and labelling it as virgin and extra virgin olive oil	Fats and oils	Fraudulent food enhancement	Product tampering	Allergenic	D
Mar-18	Meat not apt for human consumption ended up as minced meat and expiry dates were falsified	Meat and meat products	Food laundering	Non-food grade	Biological	B
Mar-18	Expired charcuterie products relabelled with a prolonged expiry date	Meat and meat products	Food laundering	Non-food grade	Biological	B
Mar-18	Meat mislabelled, defrosted, expired and contaminated and lacking required documentation for traceability	Meat and meat products	Food laundering	Non-food grade	Biological	B
Mar-18	Conventional meat labelled as organic	Meat and meat products	Fraudulent food enhancement	Product tampering	None	D
Mar-18	Charcuterie products fraudulently sold as registered trademark	Meat and meat products	Fraudulent food enhancement	Product tampering	None	D
Mar-18	Lacking traceability documentation: fish, meat, eggs, milk	Aquatic foods and products thereof	Food laundering	Unknown food grade	Biological/chemical	D
Mar-18	Lacking traceability documentation: fish, meat, eggs, milk	Meat and meat products	Food laundering	Unknown food grade	Biological/chemical	D
Mar-18	Lacking traceability documentation: fish, meat, eggs, milk	Eggs and egg products	Food laundering	Unknown food grade	Biological/chemical	D
Mar-18	Lacking traceability documentation: fish, meat, eggs, milk	Milk and milk products	Food laundering	Unknown food grade	Biological/chemical	D
Mar-18	Fake Chianti wine in powder form sold online	Wine and alcoholic beverages	Fraudulent food enhancement	Product tampering	Chemical	C
Mar-18	Cheap wine labelled as high-quality Cotes-du-Rhone wine	Wine and alcoholic beverages	Fraudulent food enhancement	Product tampering	None	D
Apr-18	Expired fish and fish without traceability documentation	Aquatic foods and products thereof	Food laundering	Non-food grade	Biological/chemical	B
Apr-18	Fish of low quality to be used in the preparation of sushi and sashimi	Aquatic foods and products thereof	Food laundering	Non-food grade	Biological	D
Apr-18	Fresh fillets actually defrosted, water and injection to increase fish weight and addition of non-authorized additives (nitrate/nitrite + ascorbic acid) for water retention and to give tuna a red colour	Aquatic foods and products thereof	Fraudulent food enhancement	Product tampering	Biological/chemical	B
Apr-18	Lack of appropriate traceability documentation	Aquatic foods and products thereof	Food laundering	Unknown food grade	None	D

Period	Issue	Product category	Food fraud category	Food fraud subcategory	Hazard	Severity category
Apr-18	Horsemeat from animals 'unfit for consumption" were sold	Meat and meat products	Food laundering	Non-food grade	Biological/chemical	B
Apr-18	Fraud in the production of Prosciutto di Parma and San Daniele	Meat and meat products	Facilitation	Facilitates record tampering	None	D
Apr-18	Europol and Interpol operation finds counterfeited and substandard foods, including selling of rotten meat, colorants, non-compliant baby foods	Meat and meat products	Fraudulent food enhancement	Product tampering	Biological/chemical	B
Apr-18	Europol and Interpol operation finds counterfeited and substandard foods, including selling of rotten meat, colorants, non-compliant baby foods	Other food/mixed	Fraudulent food enhancement	Product tampering	Biological/chemical	B
Apr-18	Chaptalization of wine	Wine and alcoholic beverages	Fraudulent food enhancement	Product tampering	None	D
May-18	Aqua cultured seas bass sold as wild caught in the Mediterranean	Aquatic foods and products thereof	Fraudulent food enhancement	Record tampering	None	D
May-18	High proportion of coffee sold as 100% Arabica contained cheaper Robusta species	Cocoa and cocoa preparations, coffee and tea	Fraudulent food enhancement	Product tampering	None	D
May-18	Olive oil missing label information, mislabelling and some oils contain a mixture of olive oils and other oils	Fats and oils	Fraudulent food enhancement	Product tampering	Allergenic	C
May-18	falsified expiring date and incorrectly labelled as organic	Meat and meat products	Food laundering	Non-food grade	Biological	C
May-18	Pasta not filed with a PDO cheese as mentioned on the label	Prepared dishes and snacks	Fraudulent food enhancement	Product tampering	None	D
May-18	Wine labels indicating the geographical origin and state's tax stamp were counterfeit	Wine and alcoholic beverages	Fraudulent food enhancement	Product tampering	None	D
Jun-18	Canning grade tuna sold as 'fresh tuna' and vegetable colourants added to increase colour	Aquatic foods and products thereof	Fraudulent food enhancement	Product tampering	Biological/chemical	C
Jun-18	Expired eggs lacking traceability information and for which the consumption deadline has been fraudulently extended	Eggs and egg products	Food laundering	Non-food grade	Biological	C
Jun-18	Spices substituted with nuts. Species used for medicinal purposed sold as edible.	Herbs and spices	Fraudulent food enhancement	Product tampering	Allergenic/chemical	B
Jun-18	Expired meat re-labelled and re-introduced into the market	Meat and meat products	Food laundering	Non-food grade	Biological	B
Jun-18	Substitution of mozzarella di bufala and other types of cheese made with buffalo milk	Milk and milk products	Fraudulent food enhancement	Product tampering	Biological	D
Jun-18	Cheese produced in Belgium sold under the guise of the famous Italian mozzarella di bufala	Milk and milk products	Fraudulent food enhancement	Product tampering	None	D
Jun-18	Conventionally produced products labelled as organic	Eggs and egg products	Fraudulent food enhancement	Record tampering	None	D

Period	Issue	Product category	Food fraud category	Food fraud subcategory	Hazard	Severity category
Jun-18	Conventionally produced products labelled as organic	Fruits and vegetables	Fraudulent food enhancement	Record tampering	None	D
Jun-18	Conventionally produced products labelled as organic	Herbs and spices	Fraudulent food enhancement	Record tampering	None	D
Jun-18	Conventionally produced products labelled as organic	Cereals and bakery products	Fraudulent food enhancement	Record tampering	None	D
Jun-18	Conventionally produced products labelled as organic	Aquatic foods and products thereof	Fraudulent food enhancement	Record tampering	None	D
Jun-18	Wine falsely declared as Verdicchio dei Castelli di Jesi DOC	Wine and alcoholic beverages	Fraudulent food enhancement	Product tampering	None	D
Jun-18	Chaptalisation of wine	Wine and alcoholic beverages	Fraudulent food enhancement	Product tampering	None	D
Jul/Aug-18	Decomposed fish from a fish shop for not fulfilling the hygienic conditions required by law	Aquatic foods and products thereof	Food laundering	Non-food grade	Biological/physical	B
Jul/Aug-18	Fish intended to be sold as fresh was rotten. Rest of stock came from Asia but labelled as Italian	Aquatic foods and products thereof	Food laundering	Non-food grade	Biological	B
Jul/Aug-18	Fish destined for canning treated with additives to mimic appearance of fresh fish	Aquatic foods and products thereof	Fraudulent food enhancement	Product tampering	Biological/chemical	C
Jul/Aug-18	Fish lacking traceability documentation and not fit for human consumption	Aquatic foods and products thereof	Food laundering	Non-food grade	Biological/chemical	B
Jul/Aug-18	Endangered Spanish eel species smuggled into Japan via China	Aquatic foods and products thereof	Food laundering	Food grade	None	D
Jul/Aug-18	Expired hams relabelled to extend their expiry date. Some already rotting	Meat and meat products	Food laundering	Non-food grade	Biological	B
Jul/Aug-18	Rotten meat stored under conditions that did not fulfil the legislated hygiene requirements	Meat and meat products	Food laundering	Non-food grade	Biological/physical	B
Jul/Aug-18	Replacement of labels to extend expiry dates, substitution of organic products by conventional, lack of hygiene and traceability of documents	Other food product / mixed	Fraudulent food enhancement	Record tampering	Biological	C
Jul/Aug-18	Expired and rotten food relabelled and intended to be offered for sale	Other food product / mixed	Food laundering	Non-food grade	Biological	B
Jul/Aug-18	Mislabelling and lack of traceability documents	Fruits and vegetables	Fraudulent food enhancement	Product tampering	Biological	D
Jul/Aug-18	Mislabelling and lack of traceability documents	Meat and meat products	Fraudulent food enhancement	Product tampering	Biological	D
Jul/Aug-18	Mislabelling and lack of traceability documents	Aquatic foods and products thereof	Fraudulent food enhancement	Product tampering	Biological	D
Jul/Aug-18	Spanish wine sold in France as French rose	Wine and alcoholic beverages	Fraudulent food enhancement	Product tampering	None	D

Period	Issue	Product category	Food fraud category	Food fraud subcategory	Hazard	Severity category
Jul/Aug-18	Cheap wine rebottled and rebranded as Pingus and Flor the Pingus, some of the most expensive Spanish wines	Wine and alcoholic beverages	Fraudulent food enhancement	Product tampering	None	D
Sep-18	Fish unsuitable for human consumption to be sold without required authorisations	Aquatic foods and products thereof	Food laundering	Unknown food grade	Biological/chemical	B
Sep-18	Lack of traceability documentation and non-respect for sanitary rules	Aquatic foods and products thereof	Food laundering	Unknown food grade	None	C
Sep-18	Rice of Asian origin falsely labelled as Italian	Cereals and bakery products	Fraudulent food enhancement	Record tampering	None	D
Sep-18	Lower quality olive oil sold as extra virgin which contained oil from other seed oils	Fats and oils	Fraudulent food enhancement	Product tampering	Allergenic	D
Sep-18	Half of olive oils sold in France are falsely labelled	Fats and oils	Fraudulent food enhancement	Product tampering	Allergenic	D
Sep-18	Lack of traceability documentation	Fruits and vegetables	Food laundering	Unknown food grade	Biological/chemical	D
Sep-18	Ham not produced according to the mandatory compliance process to be labelled as Crudo di Parma	Meat and meat products	Fraudulent food enhancement	Product tampering	None	D
Sep-18	Sold with false labels of the pecorino crotonese PDO cheese	Milk and milk products	Fraudulent food enhancement	Record tampering	None	D
Sep-18	Meat and fish not fit for human consumption	Meat and meat products	Food laundering	Non-food grade	Biological	B
Sep-18	Meat and fish not fit for human consumption	Aquatic foods and products thereof	Food laundering	Non-food grade	Biological	B
Sep-18	Poor quality wine to which synthetic aromas were suspected to be added	Wine and alcoholic beverages	Fraudulent food enhancement	Product tampering	Chemical	C
Sep-18	Sugar added before fermentation to increase alcohol content. Other chemicals including tartaric acid and sulphuric acid also stored on premises	Wine and alcoholic beverages	Fraudulent food enhancement	Product tampering	Chemical	C
Oct-18	Lack of traceability information and non-respect of the expiry date	Aquatic foods and products thereof	Food laundering	Non-food grade	Biological/chemical	C
Oct-18	Untraceable fish unfit for human consumption	Aquatic foods and products thereof	Food laundering	Non-food grade	Biological/chemical	B
Oct-18	Fish products of dubious origin without necessary traceability	Aquatic foods and products thereof	Food laundering	Unknown food grade	Biological/chemical	C
Oct-18	Untraceable fish unfit for human consumption	Aquatic foods and products thereof	Food laundering	Non-food grade	Biological/chemical	B
Oct-18	Fish dishes in restaurants did not correspond to the species indicated on the menu	Aquatic foods and products thereof	Fraudulent food enhancement	Product tampering	None	D
Oct-18	Virgin olive oils sold as extra virgin	Fats and oils	Fraudulent food enhancement	Product tampering	None	D

Period	Issue	Product category	Food fraud category	Food fraud subcategory	Hazard	Severity category
Oct-18	Replacing the expiry date label	Fruits and vegetables	Food laundering	Non-food grade	Biological	C
Oct-18	Chanterelles transported without documents of their origin and exceeding authorised quantities	Fruits and vegetables	Food laundering	Unknown food grade	Biological/chemical	D
Oct-18	Damaged food and clandestine slaughter	Meat and meat products	Food laundering	Unknown food grade	Biological/chemical	B
Oct-18	Mozzarella claimed to be produced using buffalo milk made mostly with cow milk	Milk and milk products	Fraudulent food enhancement	Product tampering	Allergenic	D
Oct-18	Lack of traceability of milk	Milk and milk products	Food laundering	Unknown food grade	Biological/chemical	D
Oct-18	Misleading information about geographical origin and restaurants selling frozen fish and fresh	Aquatic foods and products thereof	Fraudulent food enhancement	Record tampering	Biological/chemical	D
Oct-18	Misleading information about geographical origin and restaurants selling frozen fish and fresh	Aquatic foods and products thereof	Fraudulent food enhancement	Record tampering	Biological/chemical	D
Oct-18	Seasonal products without labelling from which it was possible to deduce the origin, production date, expiry date and other essential data	Meat and meat products	Food laundering	Unknown food grade	Biological	C
Oct-18	Seasonal products without labelling from which it was possible to deduce the origin, production date, expiry date and other essential data	Milk and milk products	Food laundering	Unknown food grade	Biological	C
Oct-18	Seasonal products without labelling from which it was possible to deduce the origin, production date, expiry date and other essential data	Cereals and bakery products	Food laundering	Unknown food grade	Biological	C
Oct-18	Seasonal products without labelling from which it was possible to deduce the origin, production date, expiry date and other essential data	Fats and oils	Food laundering	Unknown food grade	Biological	C
Oct-18	Fake Verdicchio, a PDO wine	Wine and alcoholic beverages	Fraudulent food enhancement	Product tampering	None	D
Nov-18	Disguising imported oil as Italian	Fats and oils	Fraudulent food enhancement	Record tampering	None	D
Nov-18	Normal onions labelled with protected PGI label	Fruits and vegetables	Fraudulent food enhancement	Product tampering	None	D
Nov-18	Imported honey mixed with honey produced in Corsica and marketed as a PDO honey	Honey and royal jelly	Fraudulent food enhancement	Product tampering	None	D
Nov-18	Meat lacking traceability of origin	Meat and meat products	Fraudulent food enhancement	Record tampering	None	D
Nov-18	Mix of cow and buffalo milk to produce mozzarella di buffala	Milk and milk products	Fraudulent food enhancement	Product tampering	Allergenic	D
Nov-18	Grana Padano and Parmiggiano sold in Canada making fraudulent use of the label 'made in Italy	Milk and milk products	Fraudulent food enhancement	Record tampering	None	D

Period	Issue	Product category	Food fraud category	Food fraud subcategory	Hazard	Severity category
Nov-18	Several irregularities, lack of hygienic conditions, Misuse of PDO labels	Other food product / mixed	Fraudulent food enhancement	Record tampering	Allergenic/biological	C
Nov-18	Various products showed labels corresponding to different PDO's which did not correspond to the identity of the product	Meat and meat products	Fraudulent food enhancement	Record tampering	Biological	D
Nov-18	Various products showed labels corresponding to different PDO's which did not correspond to the identity of the product	Milk and milk products	Fraudulent food enhancement	Record tampering	Biological	D
Nov-18	Conventional foods labelled as organic	Other food product / mixed	Fraudulent food enhancement	Product tampering	None	D
Dec-18	Cod at a premises of a company where a large amount of bleach (lime) was found	Aquatic foods and products thereof	Food laundering	Non-food grade	Biological/chemical	C
Dec-18	Fake olive oil produced by mixing capsules containing toxic substances with water to produce an oil-like liquid	Fats and oils	Fraudulent food enhancement	Product tampering	Chemical	B
Dec-18	Meat labelled as Italian actually grown in other countries	Meat and meat products	Fraudulent food enhancement	Record tampering	None	D
Dec-18	Wine with irregularities in production, lacking traceability and grape must from unknown origin	Wine and alcoholic beverages	Fraudulent food enhancement	Product tampering	Biological/chemical	D
Dec-18	Mixture of different table wines under a protected denomination of origin	Wine and alcoholic beverages	Fraudulent food enhancement	Product tampering	None	D
Dec-18	Fake Verdicchio wine, a well-known Italian PDO	Wine and alcoholic beverages	Fraudulent food enhancement	Product tampering	None	D
Jan-19	Tomatoes labelled as originating from Almeria suspected as being from Portugal	Fruits and vegetables	Fraudulent food enhancement	Record tampering	None	D
Jan-19	Meat of sick cows not apt for human consumption still exported	Meat and meat products	Food laundering	Non-food grade	Biological	B
Jan-19	Imported live animals were slaughtered but labelled as originating from Malta	Meat and meat products	Fraudulent food enhancement	Record tampering	None	D
Jan-19	PDO and PGI wine not complying with the criteria	Wine and alcoholic beverages	Fraudulent food enhancement	Product tampering	None	D
Feb-19	Expensive species replaced by cheap ones	Aquatic foods and products thereof	Fraudulent food enhancement	Product tampering	None	D
Feb-19	Commercialisation of eggs contaminated with fipronil while labels started free range	Eggs and egg products	Food laundering	Non-food grade	Chemical	B
Feb-19	Mince containing sulphite, a substance whose addition to meat is forbidden, or stored incorrectly	Meat and meat products	Fraudulent food enhancement	Product tampering	Allergenic/biological	C
Feb-19	Regular beef sold as Japanese Kobe beef	Meat and meat products	Fraudulent food enhancement	Product tampering	None	D
Feb-19	Rotten food not fit for consumption lacking labels and traceability information	Aquatic foods and products thereof	Food laundering	Non-food grade	Biological	B
Feb-19	Wine of inferior quality labelled as PGI and olive oil labelled as extra virgin not meeting required quality criteria	Wine and alcoholic beverages	Fraudulent food enhancement	Product tampering	None	D

Period	Issue	Product category	Food fraud category	Food fraud subcategory	Hazard	Severity category
Feb-19	Wine of inferior quality labelled as PGI and olive oil labelled as extra virgin not meeting required quality criteria	Fats and oils	Fraudulent food enhancement	Product tampering	None	D
Feb-19	Sugar added to wine to increase alcohol content	Wine and alcoholic beverages	Fraudulent food enhancement	Product tampering	None	D
Feb-19	Wine mislabelled as PDO and PGI	Wine and alcoholic beverages	Fraudulent food enhancement	Record tampering	None	D
Feb-19	Counterfeit trademarks and labels of a famous winery	Wine and alcoholic beverages	Fraudulent food enhancement	Product tampering	None	D
Mar-19	Did not fulfil the legal traceability requirements	Aquatic foods and products thereof	Food laundering	Unknown food grade	Biological/chemical	D
Mar-19	Kiwis sold in France as being French when actually Italian kiwis	Fruits and vegetables	Fraudulent food enhancement	Record tampering	Chemical	D
Mar-19	Genuine saffron mixed with parts of the plant not considered food, as well as other plant extracts and chemical reagents	Herbs and spices	Fraudulent food enhancement	Product tampering	Chemical/physical	B
Mar-19	Acorn shortage seemed not to influence registration numbers of Iberian pigs	Meat and meat products	Fraudulent food enhancement	Record tampering	None	D
Mar-19	New cut of chicken classified as generic product to evade import duties then sold on the EU market as chicken breast	Meat and meat products	Food laundering	Food grade	None	D
Mar-19	Grapes not filling the requirements for the production of PDO product	Soups, broths, sauces and condiments	Fraudulent food enhancement	Record tampering	None	D
Mar-19	Counterfeit trademarks and labels of a famous winery	Wine and alcoholic beverages	Fraudulent food enhancement	Product tampering	None	D
Apr-19	Sale of standard olive oil under the label extra virgin	Fats and oils	Fraudulent food enhancement	Product tampering	None	D
Apr-19	Illegal market and rotten meat lacking traceability	Meat and meat products	Food laundering	Non-food grade	Biological/chemical	B
Apr-19	Supermarket sold food with wrong labelling regarding origin and PDO labelling + hygiene	Other food product / mixed	Fraudulent food enhancement	Product tampering	Biological	C
Apr-19	Foods lacking traceability documentation and some not apt for human consumption	Nuts, nut products and seeds	Food laundering	Non-food grade	Biological/chemical/physical	C
Apr-19	Foods lacking traceability documentation and some not apt for human consumption	Herbs and spices	Food laundering	Non-food grade	Biological/chemical/physical	C
Apr-19	Foods lacking traceability documentation and some not apt for human consumption	Cereals and bakery products	Food laundering	Non-food grade	Biological/chemical/physical	C
Apr-19	Foods lacking traceability documentation and some not apt for human consumption	Honey and royal jelly	Food laundering	Non-food grade	Biological/chemical/physical	C
May-19	Cheese labelled as PGI product without fulfilling the requirements	Milk and milk products	Fraudulent food enhancement	Product tampering	Biological/chemical	D

Period	Issue	Product category	Food fraud category	Food fraud subcategory	Hazard	Severity category
May-19	Expired cheese and pesto found with falsified labels that indicated wrong origin and quality	Milk and milk products	Food laundering	Non-food grade	Biological	C
May-19	Expired cheese and pesto found with falsified labels that indicated wrong origin and quality	Soups, broths, sauces and condiments	Food laundering	Non-food grade	Biological	C
May-19	Wine not having minimum product standard required by law	Wine and alcoholic beverages	Food laundering	Non-food grade	None	D
May-19	Fake Verdicchio dei Castelli di Jesi	Wine and alcoholic beverages	Fraudulent food enhancement	Product tampering	None	D
Jun-19	Lacking traceability documentation	Aquatic foods and products thereof	Food laundering	Unknown food grade	None	D
Jun-19	Expiry date fraudulently extended in packing centre	Eggs and egg products	Food laundering	Non-food grade	Biological	C
Jun-19	Spanish strawberries sold as a French product	Fruits and vegetables	Fraudulent food enhancement	Record tampering	None	D
Jun-19	Minced meat patties mostly contained fat and skin in addition to starch and soya, which are not authorised ingredients for this type of product	Meat and meat products	Fraudulent food enhancement	Product tampering	Allergenic	C
Jun-19	Hams did not comply with the requirements to bear the PDO labels	Meat and meat products	Facilitation	Facilitates record tampering	None	D
Jun-19	Conventional products sold as organic	Other food product / mixed	Fraudulent food enhancement	Product tampering	Biological/chemical	D
Jun-19	Truffles in processed foods replaced by synthetic aroma	Prepared dishes and snacks	Fraudulent food enhancement	Product tampering	None	C
Jul/Aug-19	Olive oil labelled as organic that did not fulfil the requirements	Fats and oils	Fraudulent food enhancement	Record tampering	None	D
Jul/Aug-19	Widespread listeriosis as meat producer sold contaminated meat to a second company	Meat and meat products	Food laundering	Non-food grade	Biological	A
Jul/Aug-19	Meat conventionally produced was labelled as organic and sold in another country	Meat and meat products	Fraudulent food enhancement	Product tampering	None	D
Jul/Aug-19	Illicit trade of goods	Other food product / mixed	Food laundering	Unknown food grade	Biological/chemical	D
Sep-19	False brandy obtained from maize	Wine and alcoholic beverages	Fraudulent food enhancement	Product tampering	Allergenic	D
Sep-19	Selling seed oil as olive oil	Fats and oils	Fraudulent food enhancement	Product tampering	Allergenic	D
Sep-19	Dutch and Spanish tomatoes labelled as French	Fruits and vegetables	Fraudulent food enhancement	Record tampering	None	D
Sep-19	Increasing the weight of chicken meat with water	Meat and meat products	Fraudulent food enhancement	Product tampering	None	D

Period	Issue	Product category	Food fraud category	Food fraud subcategory	Hazard	Severity category
Sep-19	Meat sold as lamb in Spain likely from other countries	Meat and meat products	Fraudulent food enhancement	Record tampering	None	D
Sep-19	False labels for Rueda PDO wine	Wine and alcoholic beverages	Fraudulent food enhancement	Record tampering	None	D
Oct-19	Eggs labelled as organic produced by hens in cages	Eggs and egg products	Fraudulent food enhancement	Record tampering	None	D
Oct-19	Seed oil labelled as olive oil	Fats and oils	Fraudulent food enhancement	Product tampering	Allergenic	D
Oct-19	Tomatoes labelled as an Italian PDO that lacked the right certificates to prove their origin	Fruits and vegetables	Fraudulent food enhancement	Record tampering	None	D
Oct-19	Polish mushrooms sold in France as produced in France	Fruits and vegetables	Fraudulent food enhancement	Record tampering	None	D
Oct-19	Selling wine kits (powdered wine and berry derivatives) as Chianti wine	Wine and alcoholic beverages	Fraudulent food enhancement	Product tampering	Chemical	C
Oct-19	Wine labelled as Piedirosso, Aglianico del Sannio and Greco that did not fulfil the labelling requirements	Wine and alcoholic beverages	Fraudulent food enhancement	Record tampering	None	D
Nov/Dec-19	Food distributed to schools mislabelled, extra virgin olive oil as organic and frozen meat as fresh	Fats and oils	Fraudulent food enhancement	Product tampering	Allergenic/biological/physical	D
Nov/Dec-19	Food distributed to schools mislabelled, extra virgin olive oil as organic and frozen meat as fresh	Meat and meat products	Fraudulent food enhancement	Product tampering	Allergenic/biological/physical	D
Nov/Dec-19	Lack of traceability and storage without respecting hygienic requirements	Wine and alcoholic beverages	Food laundering	Non-food grade	Biological	C
Jan-20	Criminal network selling horsemeat not fit for human consumption	Meat and meat products	Food laundering	Non-food grade	Biological/chemical	B
Jan-20	Improperly stored cheese being sold in small portions as Grana Padano and Parmigiano Reggiano	Milk and milk products	Food laundering	Non-food grade	Biological/chemical	C
Jan-20	Lost cost wine being sold as a quality product for a high price, in some cases as being organic. Vinous matrix was artificially reconstructed with the addition of colouring substances and forbidden additives	Wine and alcoholic beverages	Fraudulent food enhancement	Product tampering	Chemical	D
Jan-20	Incorrectly labelled as PDO, PGI and organic	Wine and alcoholic beverages	Fraudulent food enhancement	Record tampering	None	D
Feb-20	Mixing in rotten eggs to semi-elaborated products	Eggs and egg products	Food laundering	Non-food grade	Biological/physical	B
Feb-20	Mislabelled as produced by hens grown in open air and laying date manipulated to increase durability	Eggs and egg products	Fraudulent food enhancement	Record tampering	Biological	C
Feb-20	Moroccan tomatoes relabelled as Spanish once in Spain	Fruits and vegetables	Fraudulent food enhancement	Record tampering	None	D
Feb-20	One death caused by counterfeited wine that contained high levels of the drugs MDMA and MDA	Wine and alcoholic beverages	Fraudulent food enhancement	Product tampering	Chemical	A

Period	Issue	Product category	Food fraud category	Food fraud subcategory	Hazard	Severity category
Feb-20	Counterfeited wine	Wine and alcoholic beverages	Fraudulent food enhancement	Product tampering	None	D
Feb-20	Selling young wine as more expensive	Wine and alcoholic beverages	Fraudulent food enhancement	Record tampering	None	D
Feb-20	Clandestine cantina with artificial aromas, colourants, nutrients and fermentation starters	Wine and alcoholic beverages	Fraudulent food enhancement	Product tampering	None	C
Mar-20	Lacked requested information about its origin, illegally placed back on the market after removal for non-compliance with legal hygienic requirements	Honey and royal jelly	Food laundering	Non-food grade	Biological/chemical	C
Apr-20	Labels did not reflect the origin or the way of production	Eggs and egg products	Fraudulent food enhancement	Record tampering	None	D
Apr-20	Cheese to be sold as PDO without the required PDO and other certification markings	Milk and milk products	Fraudulent food enhancement	Record tampering	None	D
May-20	Mislabelled with an extended expiry date	Eggs and egg products	Fraudulent food enhancement	Record tampering	Biological	C
May-20	Rotten meat to be distributed in some restaurants	Meat and meat products	Food laundering	Non-food grade	Biological	B
May-20	Unauthorised placing on the market	Meat and meat products	Food laundering	Unknown food grade	Chemical	B
Jun-20	Lack of traceability documentation or storing conditions not compliant with law	Aquatic foods and products thereof	Food laundering	Unknown food grade	Biological/chemical	D
Jun-20	Lacking proper documentation and masking origin	Aquatic foods and products thereof	Food laundering	Unknown food grade	None	D
Jun-20	Production of illegal cheese meant to be sold directly to the consumers without any traceability documentation	Milk and milk products	Food laundering	Unknown food grade	Biological/chemical	D
Jun-20	Imported products lacking necessary traceability documentation	Cereals and bakery products	Food laundering	Unknown food grade	Biological/chemical	D
Jun-20	Imported products lacking necessary traceability documentation	Non-alcoholic beverages	Food laundering	Unknown food grade	Biological/chemical	D
Jul/Aug-20	Iso-glucose (corn syrup) to make wine, and alcohol from cereals (as opposed to wine alcohol) to make spirits and brandy	Wine and alcoholic beverages	Fraudulent food enhancement	Product tampering	Allergenic	D
Jul/Aug-20	Lacking necessary traceability documentation and not compliant with minimal fish size	Aquatic foods and products thereof	Food laundering	Food grade	Biological/chemical	D
Jul/Aug-20	Origin of fish products changed and expiration date falsified	Aquatic foods and products thereof	Food laundering	Non-food grade	Biological/chemical	C
Jul/Aug-20	Lack of necessary traceability documentation and labels	Aquatic foods and products thereof	Food laundering	Unknown food grade	Biological/chemical	D
Jul/Aug-20	Lack of required traceability documentation	Aquatic foods and products thereof	Food laundering	Food grade	Biological/chemical	D

Period	Issue	Product category	Food fraud category	Food fraud subcategory	Hazard	Severity category
Jul/Aug-20	Lacking the necessary traceability documentation	Aquatic foods and products thereof	Food laundering	Unknown food grade	Biological/chemical	D
Jul/Aug-20	Illegally fished without any authorisation	Aquatic foods and products thereof	Food laundering	Food grade	Biological/chemical	D
Jul/Aug-20	Lack of proper traceability documentation	Eggs and egg products	Food laundering	Unknown food grade	Biological	D
Jul/Aug-20	Lacking required traceability documentation and not compliant with hygienic standards	Eggs and egg products	Food laundering	Non-food grade	Biological	C
Jul/Aug-20	Quality inferior to that claimed on the label/ labels incorrect or nutritional information was incorrect	Fats and oils	Fraudulent food enhancement	Product tampering	None	D
Jul/Aug-20	Organic almonds lacking the required traceability documentation	Nuts, nut products and seeds	Fraudulent food enhancement	Product tampering	Chemical	D
Jul/Aug-20	Lacking required traceability documentation and in many cases not compliant with hygienic standards	Meat and meat products	Food laundering	Non-food grade	Biological	C
Jul/Aug-20	Lacking required traceability documentation and in many cases not compliant with hygienic standards	Cereals and bakery products	Food laundering	Non-food grade	Biological	C
Jul/Aug-20	Lacking required traceability documentation and in many cases not compliant with hygienic standards	Other food product / mixed	Food laundering	Non-food grade	Biological	C
Jul/Aug-20	Lacking required traceability documentation and in many cases not compliant with hygienic standards	Milk and milk products	Food laundering	Non-food grade	Biological	C
Jul/Aug-20	Lacking required traceability documentation and in many cases not compliant with hygienic standards	Other food product / mixed	Food laundering	Non-food grade	Biological	C
Jul/Aug-20	Lacking required traceability documentation and in many cases not compliant with hygienic standards	Fruits and vegetables	Food laundering	Non-food grade	Biological	C
Jul/Aug-20	Counterfeiting high value wines	Wine and alcoholic beverages	Fraudulent food enhancement	Product tampering	None	D
Jul/Aug-20	Counterfeiting young wine as aged PDO's	Wine and alcoholic beverages	Fraudulent food enhancement	Product tampering	None	D
Sep-20	Lacking necessary traceability documentation and/ or not compliant with hygiene standards	Aquatic foods and products thereof	Food laundering	Non-food grade	Biological/chemical	C
Sep-20	Produced in Albania but mislabelled 'Made in Italy'	Fruits and vegetables	Fraudulent food enhancement	Record tampering	None	D
Sep-20	Lacking necessary traceability documentation and not compliant with hygiene standards	Meat and meat products	Food laundering	Non-food grade	Biological/chemical	B
Sep-20	Cheese produced illegally in unauthorised production plants with false documents	Milk and milk products	Food laundering	Unknown food grade	Biological	D
Sep-20	Lacking necessary labelling documentation	Milk and milk products	Food laundering	Unknown food grade	Biological/physical	D
Sep-20	Lacking necessary labelling documentation	Meat and meat products	Food laundering	Unknown food grade	Biological/physical	D

Period	Issue	Product category	Food fraud category	Food fraud subcategory	Hazard	Severity category
Sep-20	Lacking necessary labelling documentation	Non-alcoholic beverages	Food laundering	Unknown food grade	Biological/physical	D
Sep-20	Lacking necessary labelling documentation	Wine and alcoholic beverages	Food laundering	Unknown food grade	Biological/physical	D
Sep-20	Lacking necessary labelling documentation	Fruits and vegetables	Food laundering	Unknown food grade	Biological/physical	D
Sep-20	Lacking necessary labelling documentation	Fats and oils	Food laundering	Unknown food grade	Biological/physical	D
Sep-20	Missing any documentation and plant not compliant with legal hygiene requirements	Wine and alcoholic beverages	Food laundering	Non-food grade	Biological	C
Oct-20	Not complying with hygiene requirements and lacking the required traceability documentation	Aquatic foods and products thereof	Food laundering	Unknown food grade	Biological/chemical	C
Oct-20	Mislabelled or missing required traceability documentation	Aquatic foods and products thereof	Food laundering	Unknown food grade	Allergenic/biological/chemical	D
Oct-20	Lacking required traceability documentation	Aquatic foods and products thereof	Food laundering	Unknown food grade	None	D
Oct-20	Expiry date and lot numbers falsified	Eggs and egg products	Food laundering	Non-food grade	Biological	C
Oct-20	Conventional eggs fraudulently mislabelled as organic	Eggs and egg products	Fraudulent food enhancement	Record tampering	None	D
Oct-20	Imported outside the EU but missing any traceability documentation	Fruits and vegetables	Food laundering	Unknown food grade	Biological/chemical	D
Oct-20	Food products mislabelled as 'made in Italy' but actually imported from Africa. Crustaceans supposed to be grinded and fraudulently sold as fish derivatives	Cereals and bakery products	Fraudulent food enhancement	Product tampering	Allergenic/biological	C
Oct-20	Food products mislabelled as 'made in Italy' but actually imported from Africa. Crustaceans supposed to be grinded and fraudulently sold as fish derivatives	Aquatic foods and products thereof	Fraudulent food enhancement	Product tampering	Allergenic/biological	C
Oct-20	Lacking any traceability documentation or origin documentation	Meat and meat products	Food laundering	Unknown food grade	Biological/chemical	D
Oct-20	Lacking any traceability documentation or origin documentation	Other food product / mixed	Food laundering	Unknown food grade	Biological/chemical	D
Oct-20	Lacking any traceability documentation or origin documentation	Honey and royal jelly	Food laundering	Unknown food grade	Biological/chemical	D
Oct-20	Missing traceability documentation and against the law to store vinegar together with wine	Soups, broths, sauces and condiments	Food laundering	Unknown food grade	None	D
Oct-20	Missing traceability documentation and against the law to store vinegar together with wine	Wine and alcoholic beverages	Food laundering	Unknown food grade	None	D
Oct-20	Mislabelled and sold common wine a prestigious GI	Wine and alcoholic beverages	Fraudulent food enhancement	Product tampering	None	D

Period	Issue	Product category	Food fraud category	Food fraud subcategory	Hazard	Severity category
Nov-20	Mackerels expired in 2015	Aquatic foods and products thereof	Food laundering	Non-food grade	Biological	B
Nov-20	Lacking any traceability documentation	Aquatic foods and products thereof	Food laundering	Unknown food grade	Biological/chemical	D
Nov-20	Missing proper traceability documentation	Fruits and vegetables	Food laundering	Unknown food grade	Biological/chemical	D
Nov-20	Oregano adulterated with olive leaves or containing plant-based foreign components (e.g. Wood parts)	Herbs and spices	Fraudulent food enhancement	Product tampering	Physical	C
Nov-20	Lacking required traceability documentation/produced without authorisation	Honey and royal jelly	Food laundering	Unknown food grade	Biological/chemical	D
Nov-20	Lacking required traceability documentation/produced without authorisation	Fats and oils	Food laundering	Unknown food grade	Biological/chemical	D
Nov-20	Lacking required traceability documentation/produced without authorisation	Wine and alcoholic beverages	Food laundering	Unknown food grade	Biological/chemical	D
Nov-20	Expired and rotten food destined for the black market and mislabelled as 'produced in Peru'	Alcoholic beverages	Food laundering	Non-food grade	Biological/chemical/physical	B
Nov-20	Expired and rotten food destined for the black market and mislabelled as 'produced in Peru'	Cereals and bakery products	Food laundering	Non-food grade	Biological/chemical/physical	B
Nov-20	Expired and rotten food destined for the black market and mislabelled as 'produced in Peru'	Herbs and spices	Food laundering	Non-food grade	Biological/chemical/physical	B
Nov-20	Expired and rotten food destined for the black market and mislabelled as 'produced in Peru'	Fruits and vegetables	Food laundering	Non-food grade	Biological/chemical/physical	B
Dec-20	Fake whiskey under the name of a famous brand	Wine and alcoholic beverages	Fraudulent food enhancement	Product tampering	None	D
Dec-20	Expired fish mislabelled as fresh	Aquatic foods and products thereof	Food laundering	Non-food grade	Biological	C
Dec-20	Missing required traceability documentation	Aquatic foods and products thereof	Food laundering	Unknown food grade	Biological/chemical	D
Dec-20	Missing required traceability documentation	Aquatic foods and products thereof	Food laundering	Unknown food grade	Biological/chemical	D
Dec-20	Illegal seafood products	Aquatic foods and products thereof	Food laundering	Food grade	Biological/chemical	D
Dec-20	Tunisian pomegranates mislabelled as produced in Italy	Fruits and vegetables	Fraudulent food enhancement	Record tampering	None	D
Dec-20	Illegal meat plant and failure to respect traceability requirements	Meat and meat products	Food laundering	Unknown food grade	Allergenic/biological/chemical	D
Dec-20	Conventional pistachios labelled as organic	Nuts, nut products and seeds	Fraudulent food enhancement	Record tampering	Chemical	D
Dec-20	Legal traceability documents missing or expiry dates falsified	Aquatic foods and products thereof	Food laundering	Non-food grade	Biological/chemical	C

Period	Issue	Product category	Food fraud category	Food fraud subcategory	Hazard	Severity category
Dec-20	Missing required traceability documentation	Wine and alcoholic beverages	Food laundering	Unknown food grade	Chemical	D
Dec-20	Missing required traceability documentation	Fats and oils	Food laundering	Unknown food grade	Chemical	D
Dec-20	Illegal plant producing wine and vinegar and counterfeiting GI	Wine and alcoholic beverages	Food laundering	Unknown food grade	None	D
Dec-20	Illegal plant producing wine and vinegar and counterfeiting GI	Soups, broths, sauces and condiments	Food laundering	Unknown food grade	None	D
Dec-20	Counterfeited wines. Fraudsters also added aromas, syrups and colourants not allowed	Wine and alcoholic beverages	Fraudulent food enhancement	Product tampering	Chemical	C
Jan-21	Counterfeit rum using fake labels from three famous brands	Wine and alcoholic beverages	Food laundering	Unknown food grade	Chemical	D
Jan-21	Missing required traceability documentation	Aquatic foods and products thereof	Food laundering	Unknown food grade	Biological/chemical	D
Jan-21	Mislabelled as organic	Fruits and vegetables	Fraudulent food enhancement	Record tampering	Chemical	D
Jan-21	Imported potatoes mislabelled as origin: Bulgarian	Fruits and vegetables	Fraudulent food enhancement	Record tampering	None	D
Jan-21	Unfit for human consumption and mislabelled in terms of expiry date, origin and organic labels	Meat and meat products	Food laundering	Non-food grade	Biological	B
Jan-21	Sold without respecting traceability legislation	Meat and meat products	Food laundering	Unknown food grade	Biological/chemical	D
Jan-21	Mozzarella from buffalo's and cow's milk mislabelled as 100% buffalo mozzarella, a GI product	Milk and milk products	Fraudulent food enhancement	Product tampering	Allergenic	D
Jan-21	Low quality apple juice mislabelled as organic and containing expired fruits, mycotoxins, and unauthorised substances	Non-alcoholic beverages	Fraudulent food enhancement	Product tampering	Biological/chemical	B
Jan-21	Lacking the required documentation or label. Hygienic standards not respected	Milk and milk products	Food laundering	Unknown food grade	Biological/chemical	C
Jan-21	Lacking the required documentation or label. Hygienic standards not respected	Fruits and vegetables	Food laundering	Unknown food grade	Biological/chemical	C
Feb-21	Products with fake labels imitating another known brand	Aquatic foods and products thereof	Fraudulent food enhancement	Product tampering	Biological/chemical	D
Feb-21	Fished illegally	Aquatic foods and products thereof	Food laundering	Food grade	None	D
Feb-21	Lacking the required traceability documentation	Aquatic foods and products thereof	Food laundering	Unknown food grade	None	D
Feb-21	Lacking required traceability documentation	Prepared dishes and snacks	Food laundering	Unknown food grade	Biological/chemical	D
Feb-21	Labelled as GI but lacking any traceability information	Meat and meat products	Food laundering	Unknown food grade	Biological/chemical	D

Period	Issue	Product category	Food fraud category	Food fraud subcategory	Hazard	Severity category
Feb-21	Labelled as GI but lacking any traceability information	Milk and milk products	Food laundering	Unknown food grade	Biological/chemical	D
Feb-21	Labelled as GI but lacking any traceability information	Eggs and egg products	Food laundering	Unknown food grade	Biological/chemical	D
Mar-21	Seafood samples mislabelled and replaced with another species	Aquatic foods and products thereof	Fraudulent food enhancement	Product tampering	Allergenic/biological/chemical	D
Mar-21	No traceability documentation	Aquatic foods and products thereof	Food laundering	Unknown food grade	Biological/chemical	D
Mar-21	Fished illegally	Aquatic foods and products thereof	Food laundering	Food grade	None	D
Mar-21	Lacking any traceability documentation	Aquatic foods and products thereof	Food laundering	Unknown food grade	None	D
Apr-21	Offered for sale illegally	Aquatic foods and products thereof	Food laundering	Unknown food grade	None	D
Apr-21	Products illegally sold by the same farm mislabelled or counterfeited as Gi or lacking documentation	Fruits and vegetables	Food laundering	Unknown food grade	Biological	D
Apr-21	Products illegally sold by the same farm mislabelled or counterfeited as Gi or lacking documentation	Wine and alcoholic beverages	Food laundering	Unknown food grade	Biological	D
Apr-21	Mislabelled as 'made in Italy' but produced with tomatoes from non-EU countries	Soups, broths, sauces and condiments	Fraudulent food enhancement	Record tampering	None	D
May-21	Trading bivalves unfit for human consumption, falsifying origin of bivalves contaminated by high level of toxins and pathogens	Aquatic foods and products thereof	Food laundering	Non-food grade	Biological/chemical	B
May-21	Extra virgin olive oil containing olive oil of lower quality	Fats and oils	Fraudulent food enhancement	Product tampering	None	B
May-21	Saffron mixed with floral remains, styles and stamens, treated with unsafe colourants and mislabelled as a PDO	Herbs and spices	Fraudulent food enhancement	Product tampering	Chemical	B
May-21	Exogenous sugars and additional pigments not legally allowed	Honey and royal jelly	Fraudulent food enhancement	Product tampering	None	C
May-21	Mislabelled as regards to the origin or the GI claimed on the label	Meat and meat products	Food laundering	Unknown food grade	Biological	B
May-21	Lacking any traceability documentation	Meat and meat products	Food laundering	Unknown food grade	Biological/chemical	B
Jun-21	Alcoholic beverages with fake labels and illegally produced	Wine and alcoholic beverages	Food laundering	Unknown food grade	Chemical	D
Jun-21	Thawed tuna probably contaminated with added water and nitrites/nitrates, >12 people sick	Aquatic foods and products thereof	Fraudulent food enhancement	Product tampering	Biological/chemical	A
Jun-21	Lacking required traceability documentation	Aquatic foods and products thereof	Food laundering	Unknown food grade	Biological/chemical	D

Period	Issue	Product category	Food fraud category	Food fraud subcategory	Hazard	Severity category
Jun-21	Treatment with carbon monoxide and injection of nitrates to preserve appearance	Aquatic foods and products thereof	Fraudulent food enhancement	Product tampering	Biological	B
Jun-21	Not respecting traceability requirements	Aquatic foods and products thereof	Food laundering	Unknown food grade	Biological/chemical	D
Jun-21	Mislabelling and substitution of fish	Aquatic foods and products thereof	Fraudulent food enhancement	Product tampering	Allergenic/chemical	D
Jun-21	Immature and threatened species ready to be exported to the Asian market	Aquatic foods and products thereof	Food laundering	Food grade	None	D
Jun-21	No documentation	Aquatic foods and products thereof	Food laundering	Unknown food grade	None	D
Jun-21	Falsely labelled with GI	Cocoa and cocoa preparations, coffee and tea	Fraudulent food enhancement	Record tampering	None	D
Jun-21	Extra virgin olive oil actually of lower quality	Fats and oils	Fraudulent food enhancement	Product tampering	None	D
Jun-21	Conventional seeds mislabelled as organic	Nuts, nut products and seeds	Fraudulent food enhancement	Record tampering	Chemical	D
Jun-21	No traceability documentation and stored in poor hygiene conditions	Soups, broths, sauces and condiments	Food laundering	Non-food grade	Biological/chemical	C
Jun-21	No traceability documentation and stored in poor hygiene conditions	Fruits and vegetables	Food laundering	Non-food grade	Biological/chemical	C
Jul/Aug-21	Fake rum	Wine and alcoholic beverages	Fraudulent food enhancement	Product tampering	Chemical	D
Jul/Aug-21	Frozen fish for sushi lacking any traceability documentation and sold as fresh	Aquatic foods and products thereof	Food laundering	Unknown food grade	Biological/chemical/physical	C
Jul/Aug-21	Not respecting traceability legislation	Aquatic foods and products thereof	Food laundering	Unknown food grade	Biological/chemical	D
Jul/Aug-21	Lacking any documentation required by law	Aquatic foods and products thereof	Food laundering	Unknown food grade	Biological/chemical	D
Jul/Aug-21	Not compliant with food traceability legislation	Aquatic foods and products thereof	Food laundering	Unknown food grade	None	D
Jul/Aug-21	Imported rice falsified with the logo of a famous Australian rice brand	Cereals and bakery products	Fraudulent food enhancement	Record tampering	Chemical	D
Jul/Aug-21	Addition of other botanical parts, adulteration with dyes, mislabelling of species, inability to proof GI	Herbs and spices	Fraudulent food enhancement	Product tampering	Allergenic/chemical	B
Jul/Aug-21	Ham and loin wrongly labelled as Iberian, unfit for human consumption and falsified documents and certificates	Meat and meat products	Fraudulent food enhancement	Product tampering	Biological	C
Jul/Aug-21	Lacking any traceability documentation	Meat and meat products	Food laundering	Unknown food grade	Biological/chemical	D

Period	Issue	Product category	Food fraud category	Food fraud subcategory	Hazard	Severity category
Jul/Aug-21	Mislabelled as organic	Nuts, nut products and seeds	Fraudulent food enhancement	Record tampering	Chemical	D
Jul/Aug-21	Food products lacking the required traceability documentation, poor storing conditions	Milk and milk products	Food laundering	Non-food grade	Biological	C
Jul/Aug-21	Illicit products and criminal networks	Other food product / mixed	Food laundering	Unknown food grade	Biological/chemical	D
Jul/Aug-21	Adulterated wine mislabelled as GI, added synthetic additives	Wine and alcoholic beverages	Fraudulent food enhancement	Product tampering	Chemical	C
Sep-21	Falsified capture and transport documents	Aquatic foods and products thereof	Fraudulent food enhancement	Record tampering	Biological/chemical	C
Sep-21	No documentation	Aquatic foods and products thereof	Food laundering	Unknown food grade	Biological/chemical	D
Sep-21	Endangered species used to replace more expensive ones	Aquatic foods and products thereof	Fraudulent food enhancement	Product tampering	Biological/chemical	D
Sep-21	Lacking the required traceability documentation	Aquatic foods and products thereof	Food laundering	Unknown food grade	None	D
Sep-21	Mislabelled as 'from Italy'	Fruits and vegetables	Fraudulent food enhancement	Record tampering	None	D
Sep-21	Mislabelled as a GI	Fruits and vegetables	Fraudulent food enhancement	Record tampering	None	D
Sep-21	Spoiled meat ready to be illegally used for the production of sausages	Meat and meat products	Food laundering	Non-food grade	Biological	B
Sep-21	Lacking required traceability documentation	Meat and meat products	Food laundering	Unknown food grade	Biological	D
Sep-21	Lacking required traceability documentation	Milk and milk products	Food laundering	Unknown food grade	Biological	D
Sep-21	Falsifying labels with a GI	Soups, broths, sauces and condiments	Fraudulent food enhancement	Record tampering	None	D
Oct-21	Expired and without traceability documentation	Aquatic foods and products thereof	Food laundering	Non-food grade	Biological/chemical	D
Oct-21	Without documentation	Aquatic foods and products thereof	Food laundering	Unknown food grade	Chemical	D
Oct-21	Failure to comply with labelling rules	Aquatic foods and products thereof	Food laundering	Unknown food grade	Biological	D
Oct-21	Virgin olive oil labelled as extra virgin	Fats and oils	Fraudulent food enhancement	Product tampering	None	D
Oct-21	Honey containing added sugar syrups, did not report proper origin information, did not meet freshness standards	Honey and royal jelly	Fraudulent food enhancement	Product tampering	None	D

Period	Issue	Product category	Food fraud category	Food fraud subcategory	Hazard	Severity category
Oct-21	Low hygiene, not suitable for consumption and document not compliant with the law	Meat and meat products	Food laundering	Non-food grade	Biological/physical	C
Oct-21	Not compliant with labelling legislation or imitating geographical indications	Meat and meat products	Fraudulent food enhancement	Record tampering	None	D
Oct-21	Hiding or falsifying the origin of the animal	Meat and meat products	Fraudulent food enhancement	Record tampering	None	D
Oct-21	Expired, without any label or with incomplete/deceptive labels	Cereals and bakery products	Food laundering	Non-food grade	Biological	C
Oct-21	Expired, without any label or with incomplete/deceptive labels	Prepared dishes and snacks	Food laundering	Non-food grade	Biological	C
Oct-21	Expired, without any label or with incomplete/deceptive labels	Soups, broths, sauces and condiments	Food laundering	Non-food grade	Biological	C
Oct-21	Expired, without any label or with incomplete/deceptive labels	Wine and alcoholic beverages	Food laundering	Non-food grade	Biological	C
Oct-21	Expired, without any label or with incomplete/deceptive labels	Fruits and vegetables	Food laundering	Non-food grade	Biological	C
Oct-21	Expired, without any label or with incomplete/deceptive labels	Herbs and spices	Food laundering	Non-food grade	Biological	C
Oct-21	Food smuggled without any documentation, suspect unfit for consumption	Honey and royal jelly	Food laundering	Non-food grade	Biological	C
Oct-21	Food smuggled without any documentation, suspect unfit for consumption	Fats and oils	Food laundering	Non-food grade	Biological	C
Oct-21	Food smuggled without any documentation, suspect unfit for consumption	Nuts, nut products and seeds	Food laundering	Non-food grade	Biological	C
Oct-21	Food smuggled without any documentation, suspect unfit for consumption	Fruits and vegetables	Food laundering	Non-food grade	Biological	C
Oct-21	Food smuggled without any documentation, suspect unfit for consumption	Herbs and spices	Food laundering	Non-food grade	Biological	C
Oct-21	Contraband alcoholic beverages and food with inconsistent customs declarations	Wine and alcoholic beverages	Fraudulent food enhancement	Record tampering	None	D
Oct-21	Contraband alcoholic beverages and food with inconsistent customs declarations	Prepared dishes and snacks	Fraudulent food enhancement	Record tampering	None	D
Oct-21	Contraband alcoholic beverages and food with inconsistent customs declarations	Cereals and bakery products	Fraudulent food enhancement	Record tampering	None	D
Oct-21	Bulk wine sold with fake certification belonging to three geographical indications	Wine and alcoholic beverages	Fraudulent food enhancement	Record tampering	None	D
Nov-21	Illegal production of counterfeit liquors bearing fake labels	Wine and alcoholic beverages	Fraudulent food enhancement	Product tampering	None	D
Nov-21	Seafood without traceability documentation	Aquatic foods and products thereof	Food laundering	Unknown food grade	Biological/chemical	D

Period	Issue	Product category	Food fraud category	Food fraud subcategory	Hazard	Severity category
Nov-21	Not respecting traceability requirements	Cocoa and cocoa preparations, coffee and tea	Food laundering	Unknown food grade	None	D
Nov-21	Low quality olive oil mislabelled a organic and/or extra virgin and some cases mixed with sunflower oil	Fats and oils	Fraudulent food enhancement	Product tampering	Allergenic	D
Nov-21	Food not fit for human consumption, gaps in traceability documentation and shelf life dates falsified	Other food product / mixed	Food laundering	Non-food grade	Biological	B
Nov-21	Wine without traceability documentation and not registered within the official databases	Wine and alcoholic beverages	Food laundering	Unknown food grade	None	D
Dec-21	Illegally produced	Wine and alcoholic beverages	Food laundering	Food grade	Chemical	D
Dec-21	Seafood products either without traceability documentation or expired since 2 years	Aquatic foods and products thereof	Food laundering	Non-food grade	Biological/chemical	C
Dec-21	Illicit seafood	Aquatic foods and products thereof	Food laundering	Unknown food grade	Biological/chemical	D
Dec-21	Eggplants and olives in oil with no sanitary documentation	Fruits and vegetables	Food laundering	Unknown food grade	Biological	C
Dec-21	Food and wine with no origin on label, false/non-compliant labels or counterfeit GI	Meat and meat products	Fraudulent food enhancement	Record tampering	None	D
Dec-21	Food and wine with no origin on label, false/non-compliant labels or counterfeit GI	Wine and alcoholic beverages	Fraudulent food enhancement	Record tampering	None	D
Dec-21	Wine without any documentation and origin was uncertain	Wine and alcoholic beverages	Food laundering	Unknown food grade	None	D
Jan-22	Oat mislabelled as organic and not full compliant with the traceability requirements. Stored in unauthorised establishments	Cereals and bakery products	Fraudulent food enhancement	Record tampering	Biological/chemical	D
Jan-22	Mislabelling French wheat as Italian wheat	Cereals and bakery products	Fraudulent food enhancement	Record tampering	None	D
Jan-22	Vanilla pods, extracts, flavours and sugars non-compliant	Herbs and spices	Fraudulent food enhancement	Product tampering	None	C
Jan-22	Meat processing plant with no sanitary control in place, incorrect labels, expiration dates and traceability documents were manipulated	Meat and meat products	Food laundering	Non-food grade	Biological	C
Jan-22	Pork illegally imported from China with the risk of introducing products contaminated with African Swine Fever	Meat and meat products	Food laundering	Non-food grade	Biological	C
Jan-22	Falsifying official veterinary certificates to export chicken meat disguised as other products	Meat and meat products	Fraudulent food enhancement	Record tampering	None	D
Jan-22	Fake origin documentation / labels and not compliant with hygiene requirements	Aquatic foods and products thereof	Fraudulent food enhancement	Record tampering	Biological	C

Period	Issue	Product category	Food fraud category	Food fraud subcategory	Hazard	Severity category
Jan-22	Fake origin documentation / labels and not compliant with hygiene requirements	Fruits and vegetables	Fraudulent food enhancement	Record tampering	Biological	C
Jan-22	Fake origin documentation / labels and not compliant with hygiene requirements	Herbs and spices	Fraudulent food enhancement	Record tampering	Biological	C
Feb-22	Expired seafood kept without respecting hygiene conditions and lacking the required traceability documentation	Wine and alcoholic beverages	Fraudulent food enhancement	Record tampering	Biological/chemical	B
Feb-22	Fish with no traceability documentation	Aquatic foods and products thereof	Food laundering	Non-food grade	Biological/chemical	D
Feb-22	Fish already expired but with falsified expiry dates	Aquatic foods and products thereof	Food laundering	Unknown food grade	Biological	B
Feb-22	Seafood illicitly sold or stored	Aquatic foods and products thereof	Food laundering	Non-food grade	None	C
Feb-22	Spelt products actually contained half wheat	Aquatic foods and products thereof	Food laundering	Unknown food grade	None	C
Feb-22	Pork products with labels lacking the mandatory indication of country of origin and in poor state of conservation	Cereals and bakery products	Fraudulent food enhancement	Product tampering	Biological	D
Feb-22	Sale of meat from horses suffering with cancer and not fit for human consumption	Meat and meat products	Food laundering	Non-food grade	Biological/chemical	B
Feb-22	Milk adulterated with water	Meat and meat products	Food laundering	Non-food grade	Biological/chemical	C
Feb-22	Expired food with new falsified expiry dates	Milk and milk products	Fraudulent food enhancement	Product tampering	Biological	C
Feb-22	Expired food with new falsified expiry dates	Prepared dishes and snacks	Food laundering	Non-food grade	Biological	C
Feb-22	Products illegally carrying logos from GI	Non-alcoholic beverages	Food laundering	Non-food grade	None	D
Feb-22	Expired seafood kept without respecting hygiene conditions and lacking the required traceability documentation	Wine and alcoholic beverages	Fraudulent food enhancement	Record tampering	Biological/chemical	B
Mar-22	Smuggled alcoholic beverages	Wine and alcoholic beverages	Food laundering	Unknown food grade	None	D
Mar-22	Seafood products illegally fished or without the necessary traceability documentation, sometimes even expired	Aquatic foods and products thereof	Food laundering	Non-food grade	Biological/chemical	C
Mar-22	Tuna with no traceability documentation	Aquatic foods and products thereof	Food laundering	Unknown food grade	Biological/chemical	D
Mar-22	Illegal fish, including protected species	Aquatic foods and products thereof	Food laundering	Food grade	None	D
Mar-22	Chickens illegally farmed chicken eggs with no traceability documentation.	Eggs and egg products	Food laundering	Unknown food grade	Biological	D
Mar-22	Olive oil lacking required traceability documentation	Fats and oils	Food laundering	Unknown food grade	Chemical	D

Period	Issue	Product category	Food fraud category	Food fraud subcategory	Hazard	Severity category
Mar-22	Pulses and nuts without the required traceability documentation and hygienic deficiencies	Nuts, nut products and seeds	Food laundering	Non-food grade	Biological/chemical	C
Mar-22	Contraband food with no traceability documentation	Aquatic foods and products thereof	Food laundering	Unknown food grade	Biological	D
Mar-22	Contraband food with no traceability documentation	Cereals and bakery products	Food laundering	Unknown food grade	Biological	D
Mar-22	Contraband food with no traceability documentation	Herbs and spices	Food laundering	Unknown food grade	Biological	D
Mar-22	Several food products with labelling infractions	Other food product / mixed	Food laundering	Unknown food grade	None	D
Mar-22	Several food products with labelling infractions	Meat and meat products	Food laundering	Unknown food grade	None	D
Mar-22	Several food products with labelling infractions	Wine and alcoholic beverages	Food laundering	Unknown food grade	None	D
Mar-22	Wine adulterated with unauthorised additives and falsely labelled	Wine and alcoholic beverages	Fraudulent food enhancement	Product tampering	Chemical	C
Apr-22	Contraband alcohol	Wine and alcoholic beverages	Food laundering	Unknown food grade	None	D
Apr-22	Contraband alcoholic beverages	Wine and alcoholic beverages	Food laundering	Unknown food grade	None	D
Apr-22	Species incorrectly labelled for seafood samples	Aquatic foods and products thereof	Fraudulent food enhancement	Product tampering	Allergenic/biological/chemical	D
Apr-22	Fish juveniles and fishes without traceability documentation	Aquatic foods and products thereof	Food laundering	Unknown food grade	Biological/chemical	D
Apr-22	Contraband fish	Aquatic foods and products thereof	Food laundering	Unknown food grade	Biological	D
Apr-22	Fish illegally caught or without traceability documentation	Aquatic foods and products thereof	Food laundering	Unknown food grade	None	D
Apr-22	Eggs lacking some mandatory information on the label	Eggs and egg products	Food laundering	Unknown food grade	Biological	D
Apr-22	Clandestine production of fake olive oil and extra virgin olive oil containing a mix of other vegetable oils	Fats and oils	Fraudulent food enhancement	Product tampering	Allergenic/chemical	D
Apr-22	Fake saffron made from modified gardenia extracts	Herbs and spices	Fraudulent food enhancement	Product tampering	None	C
Apr-22	Sheep ready to be illegally slaughtered	Live animals	Food laundering	Unknown food grade	Biological	D
Apr-22	Many non-compliances for foie gras such as mislabelling. Presence of unauthorised chemicals, added water or fat content, lack of traceability documentation	Meat and meat products	Fraudulent food enhancement	Product tampering	None	C
Apr-22	Counterfeiting a Geographical indication from Sardinia	Milk and milk products	Food laundering	Unknown food grade	None	D

Period	Issue	Product category	Food fraud category	Food fraud subcategory	Hazard	Severity category
Apr-22	Falsification of expiry dates, misuse of logos from GI, mislabelling, lack of traceability documentation	Meat and meat products	Food laundering	Non-food grade	Biological	C
Apr-22	Falsification of expiry dates, misuse of logos from GI, mislabelling, lack of traceability documentation	Other food product / mixed	Food laundering	Non-food grade	Biological	C
Apr-22	Falsification of expiry dates, misuse of logos from GI, mislabelling, lack of traceability documentation	Milk and milk products	Food laundering	Non-food grade	Biological	C
Apr-22	Wine without traceability documentation, adulterated with water and sugar and with fake geographical indications	Wine and alcoholic beverages	Fraudulent food enhancement	Product tampering	None	D
Apr-22	Wine with fake Geographical Indications and without traceability documentation	Wine and alcoholic beverages	Food laundering	Unknown food grade	None	D
May-22	Defrosting and adulterating fish with unsafe substances to enhance the colour. 10 people poisoned.	Aquatic foods and products thereof	Fraudulent food enhancement	Product tampering	Biological/chemical	A
May-22	Clams without traceability documentation	Aquatic foods and products thereof	Food laundering	Unknown food grade	Chemical	D
May-22	Sea urchins illegally fished and without any traceability documentation	Aquatic foods and products thereof	Food laundering	Unknown food grade	None	D
May-22	Tuna without any traceability documentation or illegally fished	Aquatic foods and products thereof	Food laundering	Unknown food grade	None	D
May-22	Wheat seeds missing required certification, faked labels and documentation with names from protected varieties	Cereals and bakery products	Fraudulent food enhancement	Record tampering	None	D
May-22	Olive oil without traceability	Fats and oils	Food laundering	Unknown food grade	Biological/chemical	D
May-22	Non-compliant extra virgin olive oil mislabelling virgin olive oil and extra virgin and lack of traceability of documentation	Fats and oils	Fraudulent food enhancement	Product tampering	None	D
May-22	Salami without traceability documentation	Meat and meat products	Food laundering	Unknown food grade	Biological/chemical	D
May-22	Meat products lacking traceability documentation, expired or unfit for consumption. Illegal slaughterhouse	Meat and meat products	Food laundering	Non-food grade	Biological	B
May-22	Pork products with no labels or documentation	Meat and meat products	Food laundering	Unknown food grade	Biological	D
May-22	Illegal sale of veterinary medicines to increase milk production and prescriptions falsified	Milk and milk products	Facilitation	Facilitates product tampering	Chemical	B
May-22	Minor discrepancy in composition and non-compliances in nutritional declaration	Milk and milk products	Fraudulent food enhancement	Product tampering	None	C
May-22	Regular cheese misdescribed with a Geographical indication	Milk and milk products	Fraudulent food enhancement	Product tampering	None	D
May-22	Food expired, relabelled and sold by street vendors	Live animals	Food laundering	Non-food grade	Biological	C
May-22	Food expired, relabelled and sold by street vendors	Milk and milk products	Food laundering	Non-food grade	Biological	C
May-22	Food without any legal documents	Aquatic foods and products thereof	Food laundering	Unknown food grade	Biological	D

Period	Issue	Product category	Food fraud category	Food fraud subcategory	Hazard	Severity category
May-22	Wine with counterfeit geographical Indications	Wine and alcoholic beverages	Fraudulent food enhancement	Record tampering	None	D
Jun-22	Bivalves with fake packing dates or without traceability documentation	Aquatic foods and products thereof	Food laundering	Non-food grade	Biological/chemical	B
Jun-22	Seafood lacking the required traceability documentation	Aquatic foods and products thereof	Food laundering	Unknown food grade	Biological/chemical	D
Jun-22	Tuna and sea urchins without traceability documentation	Aquatic foods and products thereof	Food laundering	Unknown food grade	Biological/chemical	D
Jun-22	Illegal fishing using banned methodologies. Fishes then smuggled to Romania with fake documents	Aquatic foods and products thereof	Food laundering	Food grade	Biological	D
Jun-22	Red tuna lacking the required traceability documentation	Aquatic foods and products thereof	Food laundering	Unknown food grade	None	D
Jun-22	Rice, pasta and couscous expired since 3 years relabelled with a new expiry dates	Cereals and bakery products	Food laundering	Non-food grade	Biological	D
Jun-22	Lacking phytosanitary certificates and traceability documentation	Fruits and vegetables	Food laundering	Unknown food grade	Biological/chemical	C
Jun-22	Stealing fruits and vegetables from crop fields	Fruits and vegetables	Food laundering	Food grade	None	D
Jun-22	Fresh and frozen meat without any traceability documentation	Meat and meat products	Food laundering	Unknown food grade	Biological/chemical	D
Jun-22	Fake horse and donkey certificates to divert and sell them as horse meat in the food chain	Meat and meat products	Facilitation	Facilitates record tampering	Chemical	C
Jun-22	Mixing of mechanically separated meat into poultry sausages without declaring it	Meat and meat products	Fraudulent food enhancement	Product tampering	None	C
Jun-22	Food unfit for human consumption, without traceability data or with wrong labels	Prepared dishes and snacks	Food laundering	Non-food grade	Biological/physical	B
Jun-22	Counterfeiting low quality wine with the geographical indication of Bordeaux	Wine and alcoholic beverages	Fraudulent food enhancement	Product tampering	None	D
Jul-22	Counterfeit bottles of famous brands of wine and spirit	Wine and alcoholic beverages	Fraudulent food enhancement	Product tampering	Chemical	D
Jul-22	Seafood with no traceability documentation	Aquatic foods and products thereof	Food laundering	Unknown food grade	Biological/chemical	D
Jul-22	Red tuna lacking the required traceability documentation	Aquatic foods and products thereof	Food laundering	Unknown food grade	None	D
Jul-22	Tuna hidden from the official fishing registry	Aquatic foods and products thereof	Food laundering	Unknown food grade	None	D
Jul-22	Ice creams without traceability documentation	Ices and desserts	Food laundering	Unknown food grade	Allergenic	D
Jul-22	Bovines lacking totally or partially the required ear tag, unhygienic conditions and contaminated drinking system	Live animals	Food laundering	Unknown food grade	Biological/chemical	D

Period	Issue	Product category	Food fraud category	Food fraud subcategory	Hazard	Severity category
Jul-22	Bottles of wine with counterfeit geographical indications	Wine and alcoholic beverages	Fraudulent food enhancement	Record tampering	None	D
Aug-22	Seafood without any traceability documentation	Aquatic foods and products thereof	Food laundering	Unknown food grade	Biological/chemical	D
Aug-22	Olive oil with absence or inadequacy of labelling information, substitution without vegetable oils, or wrong geographical origins	Fats and oils	Fraudulent food enhancement	Product tampering	None	D
Aug-22	Fruits and vegetables with labels faking the origin of the products	Fruits and vegetables	Fraudulent food enhancement	Record tampering	None	D
Aug-22	Stolen cheese	Milk and milk products	Food laundering	Unknown food grade	None	D
Aug-22	Food without any traceability documentation	Meat and meat products	Food laundering	Unknown food grade	Biological	D
Aug-22	Food without any traceability documentation	Aquatic foods and products thereof	Food laundering	Unknown food grade	Biological	D
Aug-22	Food without any traceability documentation	Prepared dishes and snacks	Food laundering	Unknown food grade	Biological	D
Sep-22	Contraband wine and beer	Wine and alcoholic beverages	Food laundering	Unknown food grade	None	D
Sep-22	Seafood without the required traceability documentation	Aquatic foods and products thereof	Food laundering	Unknown food grade	Biological/chemical	D
Sep-22	Critically endangered young European glass eels ready to be smuggled	Aquatic foods and products thereof	Food laundering	Food grade	None	D
Sep-22	Wheat without traceability documentation	Cereals and bakery products	Food laundering	Unknown food grade	Chemical	D
Sep-22	Olive oil adulterated with other vegetable oils and lacking reliable traceability documentation	Fats and oils	Fraudulent food enhancement	Product tampering	Allergenic/chemical	C
Sep-22	Honey from supermarkets reported with wrong botanical origin on the label	Honey and royal jelly	Fraudulent food enhancement	Record tampering	None	D
Sep-22	Two companies accused of adulteration of wine with water and sugars, counterfeiting generic wine with labels carrying fake geographical indications, production within clandestine oenological facilities	Wine and alcoholic beverages	Fraudulent food enhancement	Product tampering	None	D
Oct-22	Contraband beer and wine	Wine and alcoholic beverages	Fraudulent food enhancement	Product tampering	None	D
Oct-22	Contraband beer and smuggled wine	Wine and alcoholic beverages	Fraudulent food enhancement	Product tampering	None	D
Oct-22	Contraband vodka and whiskey	Wine and alcoholic beverages	Fraudulent food enhancement	Product tampering	None	D

Period	Issue	Product category	Food fraud category	Food fraud subcategory	Hazard	Severity category
Oct-22	Seafood illegally fished or lacking the required traceability documentation	Aquatic foods and products thereof	Food laundering	Unknown food grade	None	B
Oct-22	Sardines without traceability documentation	Aquatic foods and products thereof	Food laundering	Unknown food grade	None	D
Oct-22	Olive oil smuggled into Italy from Tunisia	Fats and oils	Fraudulent food enhancement	Record tampering	None	D
Oct-22	Honey jars adulterated with sugar syrup, pesticide residues, GM pollen or impurities	Honey and royal jelly	Fraudulent food enhancement	Product tampering	Chemical/physical	D
Oct-22	Food from Romania not compliant with the labelling legislation	Non-alcoholic beverages	Fraudulent food enhancement	Record tampering	Biological	D
Oct-22	Food from Romania not compliant with the labelling legislation	Wine and alcoholic beverages	Fraudulent food enhancement	Record tampering	Biological	D
Oct-22	Food from Romania not compliant with the labelling legislation	Other food product / mixed	Fraudulent food enhancement	Record tampering	Biological	D
Oct-22	Food from Romania not compliant with the labelling legislation	Meat and meat products	Fraudulent food enhancement	Record tampering	Biological	D
Oct-22	Wine not corresponding to related documentation, without traceability documentation, produced in an unauthorised establishment	Wine and alcoholic beverages	Fraudulent food enhancement	Record tampering	Chemical	D
Oct-22	Common wine ready to be relabelled as products from expensive brands	Wine and alcoholic beverages	Fraudulent food enhancement	Product tampering	None	D
Nov-22	Contraband beer and wine	Wine and alcoholic beverages	Food laundering	Unknown food grade	None	D
Nov-22	Seafood illegally fished or without traceability documentation	Aquatic foods and products thereof	Food laundering	Food grade	Biological/chemical	D
Nov-22	Tuna cans of generic tuna mislabelled as a more expensive variety of tuna fish	Aquatic foods and products thereof	Fraudulent food enhancement	Product tampering	None	D
Nov-22	Plastic food contact materials containing unauthorised bamboo powder	Food contact materials	Fraudulent food enhancement	Product tampering	Chemical	C
Nov-22	Frozen Spanish pork ready to be smuggled to Switzerland	Meat and meat products	Fraudulent food enhancement	Record tampering	None	D
Nov-22	Europol and Interpol operation: fake food and alcoholic beverages	Wine and alcoholic beverages	Fraudulent food enhancement	Product tampering	Biological/chemical	D
Nov-22	Europol and Interpol operation: fake food and alcoholic beverages	Cereals and bakery products	Fraudulent food enhancement	Product tampering	Biological/chemical	D
Nov-22	Europol and Interpol operation: fake food and alcoholic beverages	Fruits and vegetables	Fraudulent food enhancement	Product tampering	Biological/chemical	D
Nov-22	Products without traceability documentation, Listeria contamination	Meat and meat products	Food laundering	Unknown food grade	Biological	B

Period	Issue	Product category	Food fraud category	Food fraud subcategory	Hazard	Severity category
Nov-22	Products without traceability documentation, Listeria contamination	Milk and milk products	Food laundering	Unknown food grade	Biological	B
Dec-22	Seafood without traceability documentation, expired or unhealthy	Aquatic foods and products thereof	Food laundering	Non-food grade	Biological/chemical	B
Dec-22	Seafood expired, counterfeit or without traceability documentation	Aquatic foods and products thereof	Food laundering	Non-food grade	Biological/chemical	C
Dec-22	Tuna without traceability documentation	Aquatic foods and products thereof	Food laundering	Unknown food grade	Biological/chemical	D
Dec-22	Fish illegally fished in a protected area	Aquatic foods and products thereof	Food laundering	Food grade	None	D
Dec-22	Non-compliances in traceability documentation	Aquatic foods and products thereof	Food laundering	Unknown food grade	None	D
Dec-22	Christmas sweets mislabelled as " artisanal'' or missing any label or traceability documentation	Cereals and bakery products	Fraudulent food enhancement	Record tampering	Biological/physical	D
Dec-22	Pomace oil mislabelled as extra-virgin olive oil	Fats and oils	Fraudulent food enhancement	Product tampering	None	D
Dec-22	Falsifying registration documents and placing horsemeat on the market not suitable for human consumption	Meat and meat products	Food laundering	Non-food grade	Biological/chemical	B
Dec-22	Without traceability documentation and with poor hygiene	Cereals and bakery products	Food laundering	Unknown food grade	Biological/physical	C
Dec-22	Without traceability documentation and with poor hygiene	Meat and meat products	Food laundering	Unknown food grade	Biological/physical	C
Dec-22	Without traceability documentation and with poor hygiene	Fruits and vegetables	Food laundering	Unknown food grade	Biological/physical	C
Jan-23	Smuggled alcohol	Wine and alcoholic beverages	Food laundering	Unknown food grade	None	D
Jan-23	Untaxed contraband vodka and whiskey from Poland	Wine and alcoholic beverages	Food laundering	Unknown food grade	None	D
Jan-23	Contraband vodka illegally imported	Wine and alcoholic beverages	Food laundering	Unknown food grade	None	D
Jan-23	Seafood without traceability	Aquatic foods and products thereof	Food laundering	Unknown food grade	Biological/chemical	D
Jan-23	Olive oil without traceability documentation	Fats and oils	Food laundering	Unknown food grade	Chemical	D
Jan-23	Contraband olive oil from Albania	Fats and oils	Food laundering	Unknown food grade	Biological	D
Jan-23	Olives without traceability documentation and suspected of being stolen	Fruits and vegetables	Food laundering	Food grade	None	D
Jan-23	Meat products without traceability documentation and serious hygienic-sanitary deficiencies	Meat and meat products	Food laundering	Non-food grade	Biological/chemical/physical	B
Jan-23	Food without traceability documentation + hygiene	Meat and meat products	Food laundering	Non-food grade	Biological	C

Period	Issue	Product category	Food fraud category	Food fraud subcategory	Hazard	Severity category
Jan-23	Food without traceability documentation + hygiene	Milk and milk products	Food laundering	Non-food grade	Biological	C
Jan-23	Food without traceability documentation + hygiene	Fruits and vegetables	Food laundering	Non-food grade	Biological	C
Feb-23	Contraband wine and beer	Wine and alcoholic beverages	Food laundering	Unknown food grade	None	D
Feb-23	Contraband alcohol	Wine and alcoholic beverages	Food laundering	Unknown food grade	None	D
Feb-23	Mussels without traceability documentation	Aquatic foods and products thereof	Food laundering	Unknown food grade	Biological/chemical	D
Feb-23	Sea urchins without traceability documentation	Aquatic foods and products thereof	Food laundering	Unknown food grade	Biological/chemical	D
Feb-23	Illegally fished	Aquatic foods and products thereof	Food laundering	Food grade	None	D
Feb-23	Illegally fished	Aquatic foods and products thereof	Food laundering	Food grade	None	D
Feb-23	Sweets without traceability documentation	Cereals and bakery products	Food laundering	Unknown food grade	Biological/physical	D
Feb-23	Frozen beef with poor traceability or double labelling	Meat and meat products	Food laundering	Unknown food grade	Biological/chemical	D
Feb-23	Unauthorised plant producing cheese	Milk and milk products	Food laundering	Unknown food grade	Biological	D
Feb-23	Contraband meat and dairy products and alcoholic beverages	Meat and meat products	Food laundering	Unknown food grade	Allergenic/biological/chemical	D
Feb-23	Contraband meat and dairy products and alcoholic beverages	Milk and milk products	Food laundering	Unknown food grade	Allergenic/biological/chemical	D
Feb-23	Contraband meat and dairy products and alcoholic beverages	Wine and alcoholic beverages	Food laundering	Unknown food grade	Allergenic/biological/chemical	D
Feb-23	Criminal organisation selling conventional products as organic	Nuts, nut products and seeds	Fraudulent food enhancement	Record tampering	None	D
Feb-23	Criminal organisation selling conventional products as organic	Fruits and vegetables	Fraudulent food enhancement	Record tampering	None	D
Mar-23	Alcoholic beverages adulterated with alcohol not meant for human consumption and containing unsafe chemicals while counterfeiting labels from famous brands	Wine and alcoholic beverages	Fraudulent food enhancement	Product tampering	Chemical	B
Mar-23	Operator not correctly registered and products without traceability documentation	Aquatic foods and products thereof	Food laundering	Unknown food grade	Biological/chemical	D
Mar-23	Illegally fished	Aquatic foods and products thereof	Food laundering	Food grade	None	D

Period	Issue	Product category	Food fraud category	Food fraud subcategory	Hazard	Severity category
Mar-23	No traceability documentation	Aquatic foods and products thereof	Food laundering	Unknown food grade	None	D
Mar-23	Wheat seeds without their intellectual Property right and without paying the companies holding the rights for those plant varieties	Cereals and bakery products	Food laundering	Food grade	None	D
Mar-23	Mixture of vegetable oils and Lampante mislabelled as virgin olive oil	Fats and oils	Fraudulent food enhancement	Product tampering	Chemical	D
Mar-23	Imported honey samples suspicious for not complying with the honey directive	Honey and royal jelly	Fraudulent food enhancement	Record tampering	Allergenic/chemical	D
Mar-23	Meat products without any label and (in some cases) expired	Meat and meat products	Food laundering	Non-food grade	Biological	C
Mar-23	Dairy products without traceability documentation	Milk and milk products	Food laundering	Unknown food grade	Allergenic/biological/chemical	D
Mar-23	Wine with counterfeit labels from a famous brand	Wine and alcoholic beverages	Fraudulent food enhancement	Product tampering	None	D
Mar-23	Counterfeit sparkling wine	Wine and alcoholic beverages	Fraudulent food enhancement	Product tampering	None	D

Supplementary Table S4. Qualitative summary table of included academic publications (n=48) taken from literature databases (sorted by year).

Reference	Issue	Product category	Food fraud category	Food fraud subcategory	Hazard	Severity category
James, 1991 [32]	Ingestion of rapeseed cooking oil, which had been adulterated with aniline	Fats and oils	Fraudulent food enhancement	Product tampering	Chemical	A
Yoshida, 1994 [33]	Consumption of contaminated cooking oil resulting from the intentional denaturation of rapeseed oil. Oil was originally intended for industrial use but was further refined and sold illegally door-to-door in Spain as inexpensive olive oil.	Fats and oils	Fraudulent food enhancement	Product tampering	Chemical	A
Bell, 1996 [34]	Adulterated rapeseed oil which has been treated with 2% aniline for industrial purposes. Oil was reprocessed to remove the aniline and sold as inexpensive olive oil	Fats and oils	Fraudulent food enhancement	Product tampering	Chemical	A
Pittet et al, 1996 [35]	Highest levels of Ochratoxin A (OTA) detected among soluble coffees. This can be explained by the fact that coffee husks are more susceptible to support mould growth and OTA production than the coffee beans themselves adulterated with coffee husks and/ or coffee parchments	Cocoa and cocoa preparations, coffee and tea	Fraudulent food enhancement	Product tampering	Chemical	B
Diggle et al, 2001 [36]	Toxic oil sold in unlabelled 5-liter plastic containers sild by door to door salesman and market traders. The oil was rapeseed oil statutorily adulterated = with 2% aniline	Fats and oils	Fraudulent food enhancement	Product tampering	Chemical	A
de la Paz, 2001 [37]	Consumption of rapeseed oil denatured with 2% aniline for industrial use, subsequently refined, and then illicitly sold as pure olive oil	Fats and oils	Fraudulent food enhancement	Product tampering	Chemical	A
Magnúsdóttir et al, 2010 [38]	1. Wine producers in Italy added methanol, a cheap alcohol used in anti-freeze and for fuel, to boost the bit of their wines	Wine and alcoholic beverages	Fraudulent food enhancement	Product tampering	Chemical	A
	2. People have used ethylene glycol to refresh legally produced wine with too little sweetness	Wine and alcoholic beverages	Fraudulent food enhancement	Product tampering	Chemical	A
Rambla-Alegre et al, 2010 [39]	Intentional illegal adulteration by a high melamine level has been discovered in food. Due to its high nitrogen level, it produces an incorrect high reading in the	Milk and milk products	Fraudulent food enhancement	Product tampering	Chemical	A

Reference	Issue	Product category	Food fraud category	Food fraud subcategory	Hazard	Severity category
	protein content measurement based on the total protein content (the Kjeldahl nitrogen determination method), that allows the final price of the product to increase.					
Arlorio et al, 2010 [40]	To increase profits, unscrupulous dealers may be tempted to add lower-priced vegetable or hazelnut oils to fresh olive oils. Hazelnut oil is often used in EVOO adulterating due to the similar chemical composition with respect to both major and minor compounds	Fats and oils	Fraudulent food enhancement	Product tampering	Allergenic	C
Spink & Moyer, 2011 [7]	1. Substituting less costly species and misrepresenting them as more expensive species. Misrepresented species may be toxic or cause allergic reaction	Other foods/mixed	Food laundering	Non-food grade	Allergenic/chemical	B
	2. Misrepresenting country of origin, ingredients etc. Allergic reactions or poisoning from toxins from banned areas (for example, ciguatoxin-prone reefs)	Other foods/mixed	Fraudulent food enhancement	Record tampering	Allergenic/chemical	B
	3. Intentional adulteration of milk with melamine	Milk and milk products	Fraudulent food enhancement	Product tampering	Chemical	A
Di Girolamo, 2015 [41]	Adulteration of EVOO with less expensive oils (e.g., peanut and corn oils) causing the introduction of seed oil-derived allergens	Fats and oils	Fraudulent food enhancement	Product tampering	Allergenic	C
Ciolacu et al, 2015 [42]	*L. monocytogenes* isolated from food products imported into the EU via the Romanian Moldavian border and sold illegally at a black market in Romania (Galati) have shown distinct virulence profiles	Other foods/mixed	Food laundering	Non-food grade	Biological	A
Oniciuc et al, 2015 [43]	Livestock-associated MRSA found in associated foods sold in a black market at an EU border	Meat and meat products	Food laundering	Non-food grade	Biological	A
Pappalardo & Ferrito, 2015 [26]	A case of monosensitivity to P. hypothalamus, unlike the more frequent and better known non-specific allergy to fish parvalbumin, was recently reported in a person with oral allergy syndrome to Pangasius but a tolerance to other fish and seafood	Aquatic foods and products thereof	Fraudulent food enhancement	Record tampering	Allergenic	A

Reference	Issue	Product category	Food fraud category	Food fraud subcategory	Hazard	Severity category
Reinholds et al, 2015 [44]	Alerts of several artificial organic colorants like azo dyes and potentially hazardous inorganic particles like lead oxides added as dense additives to increase weight or improve external appeal. Sudan dyes (I–IV) are some of the best known azo dyes	Herbs and spices	Fraudulent food enhancement	Product tampering	Chemical	B
Handford et al, 2016 [45]	1. Milk adulterated with melamine to artificially increase the protein content	Milk and milk products	Fraudulent food enhancement	Product tampering	Chemical	A
	2. Addition of water to artificially increase its volume for greater profit. The water used is sometimes contaminated	Milk and milk products	Fraudulent food enhancement	Product tampering	Biological	B
	3. Milk adulterated with salicylic acid to reduce electricity costs by extending shelf-life of milk without refrigeration	Milk and milk products	Fraudulent food enhancement	Product tampering	Chemical	B
	4. Replacement of milk fat with vegetable oils (which may be adulterated with nut oils) for economic gains	Milk and milk products	Fraudulent food enhancement	Product tampering	Allergenic	C
	5. Milk adulterated with ammonium sulphate to artificially increase the protein content	Milk and milk products	Fraudulent food enhancement	Product tampering	Chemical	B
	6. Milk adulterated with formalin which reduces electricity costs by extending the shelf-life of milk so it can be preserved for a very long time at room temperature	Milk and milk products	Fraudulent food enhancement	Product tampering	Chemical	B
	7. Milk adulterated with caustic soda (sodium hydroxide) to reduce electricity costs by extending shelf-life of milk without refrigeration	Milk and milk products	Fraudulent food enhancement	Product tampering	Chemical	B
	8. Milk adulterated with boric acid to reduce costs by extending shelf-life of milk	Milk and milk products	Fraudulent food enhancement	Product tampering	Chemical	B
	9. Milk adulterated with benzoic acid to reduce costs by extending shelf-life of milk	Milk and milk products	Fraudulent food enhancement	Product tampering	Chemical	B
	10. Milk adulterated with hydrogen peroxide to reduce electricity costs by extending shelf-life of milk without refrigeration.	Milk and milk products	Food laundering	Non-food grade	Chemical	B
	11. Increased profits by adding detergents to diluted milk to enhance cosmetic nature	Milk and milk products	Food laundering	Non-food grade	Chemical	B
	12. Milk adulterated with chlorine	Milk and milk products	Food laundering	Unknown food grade	Chemical	B

Reference	Issue	Product category	Food fraud category	Food fraud subcategory	Hazard	Severity category
	13. Milk adulterated with urea to artificially increase protein content or increase profits by blending urea with other ingredients to produce synthetic milk	Milk and milk products	Fraudulent food enhancement	Product tampering	Chemical	B
	14. Milk adulterated with carbonates and bicarbonates which act as buffers to adjust pH. This reduces costs by masking pH and acidity values of badly preserved milk to pass it off as fresh	Milk and milk products	Fraudulent food enhancement	Product tampering	Chemical	B
Abernethy et al, 2016 [46]	1. Because cow's milk is the cheapest source it is usually used as an adulterant in products made from ewe's, goat's, sheep, or buffalo milk.	Milk and milk products	Fraudulent food enhancement	Product tampering	Allergenic	C
	2. Bacterial growth in milk that has become spoiled through excessive bacterial numbers inhibited by the addition of hydrogen peroxide	Milk and milk products	Fraudulent food enhancement	Product tampering	Chemical	B
Pardo, 2016 [47]	Misdescription incidents involving escolar and oil fish (fish species that contain high levels of indigestible wax esters) when the commercialization of these species is either banned or requires specific warning labels. Escolar has been swapped for mackerel and butter fish.	Aquatic foods and products thereof	Food laundering	Non-food grade	Chemical	B
MacMahon, 2016 [48]	1. Toxic oil syndrome (TOS) caused by the ingestion of rape-seed oil denatured with aniline. The refining of aniline-containing oil produces a number of potentially toxic compounds	Fats and oils	Fraudulent food enhancement	Product tampering	Chemical	A
	2. Oil substituted by one that is intended for food use, but is simply less expensive e.g. the addition of unrefined peanut or tree nut oils (hazelnut), soybean oils	Fats and oils	Fraudulent food enhancement	Product tampering	Allergenic	C
Kounelli & Kalogianni, 2017 [49]	Milk-based products produced by goat and sheep milk adulterated with bovine milk	Milk and milk products	Fraudulent food enhancement	Product tampering	Allergenic	B
Martín-Fernández et al 2017 [50]	1. Fish species substitution for toxic or low valued fish species such as the dangerous consumption of members of the Tetraodontidae family (pufferfish)	Aquatic foods and products thereof	Fraudulent food enhancement	Product tampering	Chemical	A

Reference	Issue	Product category	Food fraud category	Food fraud subcategory	Hazard	Severity category
	2. Undeclared meat species. Transference of bovine spongiform encephalopathy, a fatal neurodegenerative disease in cattle, to humans in the form of variant Creutzfeldt Jacobs Disease	Aquatic foods and products thereof	Fraudulent food enhancement	Product tampering	Biological	B
Goggin & Murphy, 2018 [51]	Serious and urgent warning to consumers advising them to avoid red palm oil products from specific companies as they contained the illegal additive Sudan IV	Fats and oils	Fraudulent food enhancement	Product tampering	Chemical	A
Kalogianni, 2018 [25]	A variety of dairy products of various animal origin, the milk has been replaced totally or partially with cheaper milk, e.g. bovine, to earn more profit	Milk and milk products	Fraudulent food enhancement	Product tampering	Allergenic	B
Montowska & Fornal, 2018 [52]	Less-expensive proteins of non-meat origin, can be fraudulently added to meat products, to substitute more expensive meat components. Furthermore, they may contain hidden allergens	Meat and meat products	Fraudulent food enhancement	Product tampering	Allergenic	B
Van Asselt et al, 2018 [53]	Sudan dyes and para red added to chilli, curry and paprika powder to intensify and maintain the natural red colour of the spices	Herbs and spices	Fraudulent food enhancement	Product tampering	Chemical	B
Esteki et al, 2019 [54]	1. Food processing aids such as clouding agents for enhancing the appeal of a food component are used when not authorized	Fats and oils	Fraudulent food enhancement	Product tampering	Biological	B
	2. Olive oil adulterated with a lower cost substitute, such as olive oil from a different origin or any other type of oil (nut or legume oils)	Fats and oils	Fraudulent food enhancement	Product tampering	Allergenic	C
	3. Higher-value seafood substituted by more affordable species which may be associated with fish poisoning or allergens.	Aquatic foods and products thereof	Fraudulent food enhancement	Product tampering	Allergenic	B
Ellis et al, 2019 [55]	Counterfeit spirits, especially ones made from denaturants such as industrial alcohols, may have high levels of methanol. Methanol is one of the most pernicious adulterants used by individuals or groups involved in the counterfeiting of food or drink	Wine and alcoholic beverages	Fraudulent food enhancement	Product tampering	Chemical	A

Reference	Issue	Product category	Food fraud category	Food fraud subcategory	Hazard	Severity category
Valand et al, 2020 [56]	Reports of the addition pf peanuts and almonds to cumin and paprika powder in the USA and Europe	Herbs and spices	Food laundering	Food grade	Allergenic	A
Robson et al, 2020 [57]	1. Adulteration with carcasses and all body parts of animals that are suspected of being infected with transmissible spongiform encephalopathy (TSE), carcasses of animals used in experiments or contaminated parts of animals including contamination via illegal treatments, medicine use etc. and specified risk material (SRM)	Meat and meat products	Food laundering	Non-food grade	Biological	B
	2. Counterfeit products are unlikely to undergo the required standard of hygiene or quality checks. Significantly increasing the risk of food safety outbreak	Meat and meat products	Food laundering	Unknown food grade	Biological	C
	3. Illegal veterinary medicine and growth promoter use causing residues to persist through the concurrent stages of the beef supply chain	Meat and meat products	Fraudulent food enhancement	Product tampering	Chemical	B
Sohrabi et al, 2020 [58]	1. Mixing beef with cheaper meats such as chicken, horse and pork or with plant proteins such as soybean or grain derivatives	Meat and meat products	Fraudulent food enhancement	Product tampering	Allergenic	B
	2. Undesirable organs from slaughtered animals, including viscera, hyaline cartilage, udder, skin, spleen, fat, bone and central nervous tissue, replacing meat	Meat and meat products	Food laundering	Non-food grade	Biological	B
Frigerio et al, 2020 [59]	Counterfeits to insect flour with low cost (and potentially allergenic) vegetable flours like wheat and soybean	Other food product / mixed	Fraudulent food enhancement	Product tampering	Allergenic	B
Angelopoulou et al, 2021 [24]	Fraudulent adulteration of products obtained from the milk of non-bovine species e.g. Goat, sheep and buffalo (Mozzarella di Bufala) with bovine milk	Milk and milk products	Fraudulent food enhancement	Product tampering	Allergenic	B
Visciano & Schirone, 2021 [60]	1. Spanish toxic oil incident occurred in 1981 was caused by fuel oil for industrial use that was sold as olive oil for human consumption	Fats and oils	Food laundering	Non-food grade	Chemical	A
	2. The palm oil imported from Nigeria and Ghana is one of the most preferred due to	Fats and oils	Fraudulent food enhancement	Product tampering	Chemical	B

Reference	Issue	Product category	Food fraud category	Food fraud subcategory	Hazard	Severity category
	the high content of β-carotene, nevertheless it is often adulterated with Sudan IV used as hue enhancer. Such compound is used as colorant impacting a rich-red, red-orange, or yellow colour to textile and plastic products, but it is banned as food additive					
	3. The presence of horse meat containing veterinary drugs in processed food products labelled as beef	Meat and meat products	Food laundering	Non-food grade	Chemical	B
	4. Addition of methanol in wines and methanol and ethanol in alcoholic beverages	Wine and alcoholic beverages	Food laundering	Unknown food grade	Chemical	A
	5. Substitution of non-toxic fish such as monkfish or quid with others that are poisonous such as pufferfish	Aquatic foods and products thereof	Fraudulent food enhancement	Record tampering	Chemical	A
	6. Addition of cow milk for production of buffalo mozzarella or bovine whey for ricotta production declared as deriving from buffalo milk	Milk and milk products	Fraudulent food enhancement	Product tampering	Allergenic	B
	7. The addition to milk of soybean or pea powder, as inexpensive components and soy, rice and almond are intentionally added into dairy products because they are cheaper than milk proteins	Milk and milk products	Fraudulent food enhancement	Product tampering	Allergenic	B
	8. The adulteration of ground and roasted coffee by addition of other components, such as brown sugar, chicory, corn, barley, wheat, soybean, or rye, is made with the objective to obtain a less expensive final product	Cocoa and cocoa preparations, coffee and tea	Food laundering	Unknown food grade	Allergenic	B
Jurica et al, 2021 [61]	1. Adulteration of meat and seafood production involving substitution with species of low commercial value. Meat with low allergenic potential can be substituted with meat products with high allergenic potential or by adding for example soy, gluten or milk proteins	Meat and meat products	Food laundering	Food grade	Allergenic	B
	2. Liquors adulterated with methanol	Wine and alcoholic beverages	Fraudulent food enhancement	Product tampering	Chemical	A

Reference	Issue	Product category	Food fraud category	Food fraud subcategory	Hazard	Severity category
	3. Wine which contained high levels of amphetamine-like stimulants	Wine and alcoholic beverages	Food laundering	Non-food grade	Chemical	A
	4. Meat intended to be sold to schools, restaurants and hotels contaminated with salmonella	Meat and meat products	Food laundering	Non-food grade	Biological	B
	5. Tuna treated with nitrates an nitrites to give it a red colour	Aquatic foods and products thereof	Food laundering	Non-food grade	Chemical	B
	6. Mixing peanuts with hazelnuts and selling it as hazelnut for economic gain	Nuts, nut products and seeds	Fraudulent food enhancement	Product tampering	Allergenic	B
Vindigni et al, 2021 [62]	1. Tetrodotoxin occurs mainly in members of the family Tetraodontidae (puffer fish), which can replace monkfish and ciguatera linked consumption of mislabelled imported tropic fish, mostly snappers and groupers	Aquatic foods and products thereof	Fraudulent food enhancement	Product tampering	Chemical	A
	2. Mislabelled tuna and salmon; presence of mercury and organochlorine compounds	Aquatic foods and products thereof	Fraudulent food enhancement	Product tampering	Chemical	B
	3. Generic hake substituted with generic cod or pollock	Aquatic foods and products thereof	Fraudulent food enhancement	Product tampering	Allergenic	B
	4. Escolar (Lepidocybium flavobrunneum) substituted with other generic escolar fish. Risk of gastrointestinal illness, fat esters causing keriorrhea of varying exigency	Aquatic foods and products thereof	Fraudulent food enhancement	Product tampering	Chemical	B
	5. Sablefish (Anoplopoma fimbria) substituted with Patagonian and Antarctic toothfish, species which are often infected with zoonotic species of Anisakis and A. simplex. Acute/chronic abdominal syndrome if consumed raw	Aquatic foods and products thereof	Fraudulent food enhancement	Product tampering	Biological	B
	6. Wreckfish (P.americanus), Grouper hybrid (E. fuscoguttatus lanceolatus), Hapuku wreckfish (P. oxygeneios) substituted with Nile tilapia (O. niloticus) or Tilapia (Oreochromis spp.). Risk for fish-borne zoonotic parasites	Aquatic foods and products thereof	Fraudulent food enhancement	Product tampering	Biological	B

Reference	Issue	Product category	Food fraud category	Food fraud subcategory	Hazard	Severity category
	7. Dactylopteridae or generic puffer fish substituted with silver-cheeked toadfish (Lagocephalus sceleratus)	Aquatic foods and products thereof	Fraudulent food enhancement	Product tampering	Chemical	B
	8. Farmed salmon substituted with wild caught salmon. Presence of organochlorine compounds which are considered carcinogenic and cause negative neurobehavioral, immune or endocrine effects	Aquatic foods and products thereof	Fraudulent food enhancement	Product tampering	Chemical	B
	9. Swordfish (Xiphias gladius) substituted with Shortfin mako (Isurus oxyrinchus), Porbeagle (Lamna nasus), Thresher sharks (Alopias spp.), Blue shark (prionace glauca) OR Atlantic bluefin tuna (Thunnus thynnus) substituted with Yellowfin tuna (Thunnus albacares), Pacific bluefin tuna (Thunnus orientalis), Bigeye tuna (Thunnus obesus) OR Tobiko/flying fish egg (Hirundichthys affinis) substituted with Bony flying fish (Hirundichthys oxycephalus), Coromandel flying fish (Hirundichthys Coromandelensis), Capelin (Mallotus villosus) OR Ikura/salmon eggs substituted with Chum salmon (Oncorhynchus keta)	Aquatic foods and products thereof	Fraudulent food enhancement	Product tampering	Allergenic	B
Robson et al, 2021 [63]	1. Azo dye E110 (Sunset yellow) used to conceal pork labelled as beef. Dyes also used to conceal other beef crimes such as expired products and increase the attractiveness of a product.	Meat and meat products	Fraudulent food enhancement	Record tampering	Chemical	B
	2. Consuming expired meat products due to changing of expiry date or sale of spoiled products	Meat and meat products	Food laundering	Non-food grade	Biological	C
	3. Consuming meat with illegal veterinary medicines and growth promoters especially in the form of antibiotics	Meat and meat products	Fraudulent food enhancement	Product tampering	Chemical	B
	4. Use of forbidden preservatives such as formaldehyde and sodium formaldehyde in food	Meat and meat products	Fraudulent food enhancement	Product tampering	Chemical	B
	5. Non meat substitutions used as bulking agents which have allergenic potential e.g. soya, whey and gluten	Meat and meat products	Fraudulent food enhancement	Product tampering	Allergenic	B

Reference	Issue	Product category	Food fraud category	Food fraud subcategory	Hazard	Severity category
	6. Product extension using Specific risk material (SRMs) which consist of materials such as the brain Nd spinal cord. Risk if this is from an animal infected with TSE	Meat and meat products	Food laundering	Non-food grade	Biological	B
Negi et al, 2021 [64]	1. Cumin and black cumin adulterated with peanut butter powder/shell	Herbs and spices	Fraudulent food enhancement	Product tampering	Allergenic	B
	2. Cinnamon adulterated with aromatized and powdered beechnut husk and hazelnut	Herbs and spices	Fraudulent food enhancement	Product tampering	Allergenic	B
	3. Chilli powder adulterated with brick/talc powder, Sudan 1&4, Para red, Rhodamine B, Orange 2	Herbs and spices	Fraudulent food enhancement	Product tampering	Chemical	B
	4. Chinese star anise adulterated with Japanese star anise	Herbs and spices	Food laundering	Non-food grade	Chemical	A
	5. Turmeric adulterated with lead chromate, chalk powder, Curcuma zedoaria and C. malabarica	Herbs and spices	Food laundering	Non-food grade	Chemical	B
	6. Saffron adulterated with dye Acid orange II, mentanil yellow, Sudan I, Ponceau 4R, Ponceau 6R	Herbs and spices	Fraudulent food enhancement	Product tampering	Chemical	B
Esteki et al, 2021 [65]	1. Nut powders, due to their high prices, are target of illegal practices such as mixing with cheaper nuts such as peanuts.	Nuts, nut products and seeds	Fraudulent food enhancement	Product tampering	Allergenic	B
	2. Spices, herbs and their products adulterated with artificial organic colourants such as azo dyes	Herbs and spices	Fraudulent food enhancement	Product tampering	Chemical	B
Kumari et al, 2021 [66]	Among the synthetic adulterants artificial dyes are most commonly used to adulterate powdered saffron	Herbs and spices	Fraudulent food enhancement	Product tampering	Chemical	B
Manning & Kowalska, 2021 [67]	Adulteration of alcohol (alcohol liqueurs, wine etc.) with the non-disclosed use of ethylene glycol and methanol to fortify (raise the alcohol level) and/or improve taste	Wine and alcoholic beverages	Fraudulent food enhancement	Product tampering	Chemical	A
Marchetti et al, 2021 [68]	Fraudulent incorporation of less expensive bovine milk into buffalo dairy	Milk and milk products	Fraudulent food enhancement	Product tampering	Allergenic	B
Erasmus et al, 2021 [69]	Adulteration of turmeric with the vibrant yellow, toxic and carcinogenic compound lead chromate	Herbs and spices	Fraudulent food enhancement	Product tampering	Chemical	B
Caldara et al, 2022 [70]	Melamine added intentionally to milk in order to mimic protein-rich milk products	Milk and milk products	Fraudulent food enhancement	Product tampering	Chemical	A

Reference	Issue	Product category	Food fraud category	Food fraud subcategory	Hazard	Severity category
Cottenet & Blancpain, 2022 [71]	Food adulteration and tampering of vegan products and vegan shrimps with real shrimp (by-)products or crustaceans	Aquatic foods and products thereof	Food laundering	Food grade	Allergenic	B
Cottenet et al, 2022 [72]	Undeclared celery detected in samples of thyme and parsley and peanut was reported in a ginger sample	Herbs and spices	Fraudulent food enhancement	Product tampering	Allergenic	B
Lawrence et al, 2022 [73]	1. Accidental allergenic exposure due to mislabelling of species	Aquatic foods and products thereof	Fraudulent food enhancement	Product tampering	Allergenic	A
	2. Illegally importation of fish that have been smoked using traditional methods can present a public health risk due to unsafe levels of polycyclic aromatic hydrocarbons	Aquatic foods and products thereof	Food laundering	Non-food grade	Chemical	B
Mottola et al, 2022 [74]	Fish burgers containing undeclared presence of molluscs, swine DNA and the inclusion of undeclared taxa potentially causing allergies	Aquatic foods and products thereof	Fraudulent food enhancement	Product tampering	Allergenic	B
Piredda et al, 2022 [27]	1. Traces of Tetraodontidae found in surimi samples	Aquatic foods and products thereof	Fraudulent food enhancement	Product tampering	Chemical	B
	2. Processed seafood samples revealed the unreported presence of molluscs	Aquatic foods and products thereof	Food laundering	Food grade	Allergenic	A

Supplementary Table S5. Qualitative summary table of included criminal investigation reports (n=31) from the NVWA-IOD.

Case nr	Issue	Product category	Food Fraud category	Food Fraud subcategory	Hazard	Severity category
1	Knowingly selling unsuitable products (contaminated sibutramine)	cocoa and cocoa preparations, coffee and tea	Fraudulent food enhancement	Product tampering	Chemical	A
2	Knowingly selling unsuitable product (contamination with salmonella)	eggs and egg products	Facilitation	Facilitates food laundering	Biological	A
3	Knowingly selling unsuitable product (salmonella contamination) and not having traceability information	eggs and egg products	Food laundering	Non-food grade	Biological	A
4	Knowingly selling unsuitable products (packaging with glass shards)	herbs and spices	Food laundering	Non-food grade	Physical	A
5	Knowingly selling unsuitable product (microbiological contaminations, neglecting hygienic procedures)	meat and meat products	Food laundering	Non-food grade	Biological	A
6	Knowingly selling unsuitable product (excess mercury levels)	Aquatic foods and products thereof	Facilitation	Facilitates food laundering	Chemical	B
7	Illegally caught products from banned location	Aquatic foods and products thereof	Food laundering	Unknown food grade	Chemical	C
8	Knowingly selling unsuitable products (contamination with aflatoxin)	cereals and bakery products	Food laundering	Non-food grade	Chemical	B
9	Knowingly selling unsuitable product (contamination with fipronil)	eggs and egg products	Food laundering	Non-food grade	Chemical	B
10	Forging laboratory results, hiding MRL exceedance for mercury, pesticides in various products	fats and oil / herbs and spices	Facilitation	Facilitates food laundering	Biological/chemical	B

Case nr	Issue	Product category	Food Fraud category	Food Fraud subcategory	Hazard	Severity category
11	Knowingly selling unsuitable products (contamination with salmonella, aflatoxin, pesticide)	fruits and vegetables / herbs and spices	Food laundering	Non-food grade	Biological/chemical	B
12	Knowingly selling unsuitable products (contamination with aflatoxin)	herbs and spices	Food laundering	Non-food grade	Chemical	B
13	Knowingly selling unsuitable product (microbiological contaminations)	meat and meat products	Food laundering	Non-food grade	Biological	B
14	Addition of undeclared sulphite to meat	meat and meat products	Fraudulent food enhancement	Product tampering	Allergenic	B
15	Knowingly selling unsuitable product (glass shards in packaging)	other food product / mixed	Food laundering	Non-food grade	Physical	B
16	Knowingly selling unsuitable products (contaminated with aflatoxin)	nuts, nut products and seeds	Food laundering	Non-food grade	Chemical	B
17	Changing best-before dates	alcoholic beverages	Fraudulent food enhancement	Record tampering	None	D
18	Forge origin description (sell Irish oysters as French)	Aquatic foods and products thereof	Fraudulent food enhancement	Record tampering	None	D
19	Forged traceability certificates	Aquatic foods and products thereof	Food laundering	Food grade	None	D
20	Selling chemical pesticide (fipronil) as natural cure against poultry illness	eggs and egg products	Facilitation	Facilitates product tampering	Chemical	B
21	Falsification of traceability codes, selling conventional eggs as organic, and diverse administrative falsifications	eggs and egg products	Food laundering	Unknown food grade	None	D
22	Forging export certificates to avoid import tariffs	meat and meat products	Fraudulent food enhancement	Record tampering	None	D
23	Illegal butchering	meat and meat products	Food laundering	Unknown food grade	Biological/chemical	C

Case nr	Issue	Product category	Food Fraud category	Food Fraud subcategory	Hazard	Severity category
24	Trading unsafe byproducts for human consumption using falsified product descriptions	meat and meat products	Food laundering	Unknown food grade	Biological/chemical	C
25	Administering antibiotics without medical need	meat and meat products	Fraudulent food enhancement	Product tampering	Chemical	C
26	Selling products with production dates in the future	meat and meat products	Fraudulent food enhancement	Record tampering	Biological	C
27	Concealing non-compliance of maximal transport times	meat and meat products	Fraudulent food enhancement	Record tampering	None	D
28	Laboratory concealed positive tests for salmonella in poultry samples	eggs and egg products	Facilitation	Facilitates food laundering	Biological	C
29	Manipulate microbiological safety assessments during production	meat and meat products	Food laundering	Unknown food grade	Biological	C
30	Selling regular butter as premium quality butter	milk and milk products	Fraudulent food enhancement	Product tampering	None	D
31	Selling products destined for destruction (for quality and esthetical reasons, no known hazards)	milk and milk products	Food laundering	Unknown food grade	Biological	C
